# Stepwise Threshold Clustering: A New Method for Genotyping MHC Loci Using Next-Generation Sequencing Technology

**DOI:** 10.1371/journal.pone.0100587

**Published:** 2014-07-18

**Authors:** William E. Stutz, Daniel I. Bolnick

**Affiliations:** 1 Department of Ecology and Evolutionary Biology, University of Colorado, Boulder, Colorado, United States of America; 2 Howard Hughes Medical Institute & Section of Integrative Biology, University of Texas at Austin, Austin, Texas, United States of America; University of Calgary, Canada

## Abstract

Genes of the vertebrate major histocompatibility complex (MHC) are of great interest to biologists because of their important role in immunity and disease, and their extremely high levels of genetic diversity. Next generation sequencing (NGS) technologies are quickly becoming the method of choice for high-throughput genotyping of multi-locus templates like MHC in non-model organisms.Previous approaches to genotyping MHC genes using NGS technologies suffer from two problems:1) a “gray zone” where low frequency alleles and high frequency artifacts can be difficult to disentangle and 2) a similar sequence problem, where very similar alleles can be difficult to distinguish as two distinct alleles. Here were present a new method for genotyping MHC loci – Stepwise Threshold Clustering (STC) – that addresses these problems by taking full advantage of the increase in sequence data provided by NGS technologies. Unlike previous approaches for genotyping MHC with NGS data that attempt to classify individual sequences as alleles or artifacts, STC uses a quasi-Dirichlet clustering algorithm to cluster similar sequences at increasing levels of sequence similarity. By applying frequency and similarity based criteria to clusters rather than individual sequences, STC is able to successfully identify clusters of sequences that correspond to individual or similar alleles present in the genomes of individual samples. Furthermore, STC does not require duplicate runs of all samples, increasing the number of samples that can be genotyped in a given project. We show how the STC method works using a single sample library. We then apply STC to 295 threespine stickleback (*Gasterosteus aculeatus*) samples from four populations and show that neighboring populations differ significantly in MHC allele pools. We show that STC is a reliable, accurate, efficient, and flexible method for genotyping MHC that will be of use to biologists interested in a variety of downstream applications.

## Introduction

The major histocompatibility complex (MHC) is a genomic region (or set of regions) unique to vertebrates that contains genes crucial for the proper functioning of the adaptive immune system. Of particular interest are the MHC class I and class II loci, which encode cell surface receptors that bind and present antigens (both self and non-self derived) to immune-effector cells [1], [2]. The resulting interaction between MHC receptors, antigens, and T-cells leads to both self-tolerance via negative selection on auto-reactive T cell variants and to activation of cell-mediated immune responses when antigens are non-self derived peptides. Consequently, MHC loci are of great interest in both the study of pathogen resistance and in the study of autoimmune disorders. Both MHC class I and class II loci are noteworthy because of their diversity within and among individuals [3]–[6], and MHC loci have served as a model genetic system for exploring questions about the selective mechanisms maintaining genetic diversity in natural populations [7]–[14]. Understanding the causes and consequences of MHC variation also has strong implications for biologists interested in the evolution and epidemiology of infectious disease [15]–[17] and the conservation of endangered populations [18], [19]. In addition to its immunological importance, variation at MHC loci has also been shown to influence mate-choice decisions in many animals, allowing the discrimination of related and unrelated individuals and of immunologically compatible and incompatible mates [20], [21]. Correctly assessing genetic variation at MHC loci is likely to remain a key component of future research, both in basic sciences like immunology, ecology, evolution, and behavior, as well as in translational research such as finding the genetic basis of various immune disorders and wildlife diseases.

Although high-throughput, locus specific methods for genotyping of human MHC (HLA) loci have recently been made available [22], [23], accurately genotyping MHC loci in non-model organisms remains a complicated challenge [24]. The biggest barriers to genotyping MHC genes occur at the PCR stage, because the MHC genes that encode the antigen binding regions often exist in multiple paralogous copies within genomes [2], [25]–[27], making traditional cloning followed by Sanger sequencing problematic. Ideally, a different pair of forward and reverse PCR primers would be used to individually amplify each MHC locus before sequencing, allowing for the unambiguous characterization of individuals as hetero- or homozygous at each paralogous copy [28]–[30]. In practice, a locus by locus approach is usually not feasible, for several reasons. First, MHC loci often share allelic lineages (groups of similar alleles that are highly divergent from other such groups) which can persist even beyond speciation events [31]–[33]. Second, different MHC loci can exhibit substantial sequence similarity due to the high rates of inter-locus recombination and gene conversion [34]–[36]. Third, many MHC gene duplications are of recent origin, making it less likely that there will be fixed sequence differences between paralogs where primers might be placed [25], [32], [37]. Combined, these factors can make it very difficult to find unique primer pairs that amplify all alleles at a single MHC locus. In practice, the only option is to use primer pairs conserved across all the MHC loci of interest (i.e. class I or class II), meaning that all the MHC alleles present within a given individuals' genome are amplified simultaneously [24]. Further challenges to genotyping MHC in non-model organisms are presented by the possible presence of pseudo-genes, loci that amplify at lower efficiency, and variation in the number of paralogs between species, populations, and individuals [16], [24], [25].

Given these challenges, sequencing MHC loci in non-model organisms has, until recently, been accomplished by 1) extensive bacterial cloning and direct sequencing [33] or 2) conformation based detection methods [24], [38]–[41]. The latter approaches rely on running PCR-amplified sequences through a charged gel matrix or capillary sequencer to identify alleles based on differences in DNA strand mobility. The main advantage of conformation based approaches (once perfected) is that many (10 s–100 s) individuals can be genotyped more quickly and cheaply relative to cloning and sequencing. The disadvantages are that (1) these methods often take significant time and effort to begin work in new systems (2) co-amplifying alleles are not always distinguishable from one another, (3) amplification bias can cause alleles to be missed, and (4) nucleotide sequences are not obtained without further sequencing, adding significant time and expense to fully characterize nucleotide variation [24], [42]. Cloning, while not being subject to the same disadvantages as conformation based approaches, can require a very large number of clones to be sequenced for each individual when allelic diversities are high, making it both cost and labor intensive whenever multiple individuals need to be genotyped. Cloning can also introduce additional sequence artifacts due to mismatch repair of heteroduplex molecules in the cloning process [43].

Given these limitations, researchers have recently taken advantage of next generation sequencing (NGS) technologies to genotype MHC loci [44]–[53]. NGS technologies allow researchers to directly sequence individual PCR amplicons, performing the equivalent of millions of cloning and sequencing reactions in a single sequencing run. Coupled with multiplexing of many barcoded samples, next-generation sequencing can allow for the complete sequencing of hundreds of individuals simultaneously [45], [54], [55]. Additionally, recent studies have shown that both cloning and conformation based approaches can significantly underestimate MHC diversity when compared to NGS approaches [49], [53], suggesting that the increased read depth of NGS allows for the identification of rare or less-efficiently amplified MHC alleles.

Next-generation sequencing has some drawbacks, however. Most notable, error rates for NGS are higher than Sanger sequencing. For example, 454 sequencing is subject to extensive homopolymer over- and underscoring [56], whereas Illumina sequencing is prone to substitution errors, with different types of substitutions (i.e. A to C) being more common than others [57]. Additionally, all next-generation sequencing approaches are still subject to artifacts generated during PCR. Of those, PCR chimeras [42], [58] are the most problematic for MHC studies because they can be indistinguishable from naturally occurring intra and inter-locus recombinants, though they will typically occur at much lower frequencies [49], [53]. Like previous approaches, using NGS technologies does not allow one to assign alleles to specific loci or to determine zygosity across loci. Cost is also a major issue (although prices for next generation sequencing technologies decrease every year) and forces researchers to carefully balance the desire to obtain genotypes for many individuals within a single sequencing run versus the desire to increase the read coverage per individual [45], [46] and to run duplicates of every sample to confirm genotypes [53].

Recently developed NGS-based approaches for genotyping MHC in non-model organisms [44], [45], [49], [53] have all adopted an approach carried over from cloning and sequencing. These approaches attempt to classify each unique sequence returned in an NGS run as either a “true” allelic sequence or as an “artifactual” one (a sequence containing errors). A given individual's MHC genotype then consists of all the unique true allelic sequences among all the sequences obtained for that individual in a given sequencing run. Although these approaches differ in the exact details, they all rely on two key assumptions: that sequences corresponding to alleles will be overrepresented relative to sequences corresponding to artifacts and that artifactual sequences will tend to be more similar to true allele sequences than true allelic sequences will be with each other [53]. Given these assumptions, recently developed approaches proceed via some combination of 1) filtering out low quality or obvious artifacts (i.e. sequences that are too short or long) 2) applying threshold criteria to sequences based on their frequency of occurrence (read depth) within an entire library or within a single sample library 3) applying criteria based on sequence similarity to differentiate artifacts from alleles, and 4) validating allele assignments using duplicate PCRs of the same sample.

Existing methods suffer from some inadequacies resulting from violations of the two assumptions listed above. The assumption that true allelic sequences will be more frequent than artifactual sequences will not always hold. Some true allelic sequences will be represented by relatively few reads in the sequencing run, either from stochastic sampling from the sample library (i.e from the larger pool of PCR amplicons subsequently selected for sequencing) or because some alleles amplify at low-efficiency relative to other alleles [53]. Additionally, some artifacts can be represented by a relatively large number of reads for a number of reasons. The occurrence of errors at early stages of PCR (and propagated by subsequent PCR cycles), the tendency of particular platforms to produce errors at certain points along a sequence (i.e. over and under-calling of homopolymer sequences), and the stochastic over-sampling of amplicons containing errors before sequencing can all produce a relatively large number of reads representing a single artifactual sequence. Consequently, there is a gray zone of moderately common sequences including both low-frequency true alleles and high-frequency artifacts [46]. Within this gray zone, applying a conservative frequency cut-off will increase the occurrence false-negatives (alleles being classified as artefacts) while more lenient thresholds will increase the number of false positives (artefacts being classified as alleles).

The second assumption – that artifacts will be more similar to alleles than alleles are to each other – will obviously be violated whenever two alleles are relatively similar (i.e. <2 base pairs different). The first methods to apply next-generation sequencing to MHC genotyping either ignored sequence similarity altogether [45], or assumed that less frequent sequences that were nearly identical (<3 bp difference) to more frequent sequences were artifacts [44]. These shortcuts will necessarily lead to some instances where true alleles are classified as artifacts merely because they are similar to other true alleles, resulting in an increase in false negatives.

Recently, Sommer et al. [53] proposed to address both the gray zone problem and the similar sequence problem by requiring that every sample be run in duplicate (i.e. two separate PCRs). This strategy works because, while a given artifact may be relatively common (i.e. fall in the gray zone) in one duplicate, it would be unlikely to be common in both duplicates. Similarly, a true allelic sequence that appears at relatively low frequency in one duplicate is unlikely to do so in another (unless the low frequency is due low amplification efficiency and not stochastic sampling of PCR amplicons). For much the same reason, two similar sequences are likely to appear at high frequencies in both duplicates if both are alleles, whereas if one is an artifact it should appear at much lower frequency in at least one if not both duplicates. Although effective, there is one main drawback to running every sample in duplicate. Sample duplication reduces by half the number of samples that can be genotyped in a given sequencing run or for a given amount of sequencing money. This cost-benefit trade off may not make fiscal sense for many researchers, for example those interested in characterizing MHC diversity across many populations or who wish to have increased statistical power (by genotyping more samples) to detect the fitness effects of individual alleles within populations.

To address the problems described above we present a new method for genotyping MHC using next-generation sequencing technologies that we call Stepwise Threshold Clustering (STC). STC takes a fundamentally different approach to genotyping MHC loci than have previously published methods [44], [45], [53]. Rather than applying frequency or similarity criteria to determine the allelic classification (true or artifact) of individual sequences, STC uses a clustering algorithm to group sequences into clusters based on sequence similarity. Crucially, unlike previous approaches, the identity of true alleles is determined on a cluster by cluster basis rather than on a sequence by sequence basis. STC is designed specifically for situations where multiple loci are co-amplified and where multiple copies of the same allelic sequence may be amplified in a single sample. STC also does not assume equal amplification efficiencies for all alleles. The method is designed to be applicable to sequence data from any NGS sequencing platform, and can be applied to any number of samples for which MHC data have been recorded. Importantly, it does not rely on including duplicate PCRs for each sample, making it comparatively cost effective.

In this paper, we first outline the STC method in detail, showing how it works by applying it to reads generated from a single sample. We then applied STC to genotype MHC class IIβ loci in 295 stickleback fish (*Gasterosteus aculeatus*) collected from two sets of paired population inhabiting different freshwater habitats (one lake and one stream population per pair). Using the sequencing results obtained by STC, we are able to show that neighboring lake and stream populations differ significantly in their multi-locus MHC genotypes, and, using the increased power afforded by sampling so many individuals, we show that many alleles are significantly more frequent in one habitat versus another when comparing paired populations. We verify the STC method by including results from six duplicate samples and by cloning and sequencing a small subset of individual samples. Lastly, we discuss the a number issues arising from the analysis including variation in amplification efficiency, minimal sample library sizes necessary for genotyping, and the applicability to non-pyrosequencing platforms.

## Overview of Stepwise Threshold Clustering (STC)

Although STC relies on the same two assumptions about the frequency and similarity of allelic and artifactual sequences that previous methods do, it does not attempt to ascertain allelic status on a sequence by sequence basis. Rather, STC is based on the proposition that, for a single individual with N alleles, the sequences generated for that individual can be grouped into approximately N clusters of similar sequence reads. This is because, with the exception of PCR chimeras, artifactual sequences will tend to be minor deviations from the sequences of true alleles. The approach taken by STC is to identify those N clusters for each individual sample, and to determine the identities of the true alleles from those N clusters.

At the heart of the STC method is an algorithm that processes reads from each sample through successive rounds of clustering using increasingly stringent levels of sequence similarity. After each round, the resulting clusters are tested against two criteria to determine whether they correspond to one, and only one, of the original N alleles for that sample. First, a cluster must must contain enough reads relative to the sample library size (i.e. be large enough), which ensures rare but highly divergent sequences (e.g. PCR chimeras) are not counted as true alleles. Second, because the majority of reads in a given run are expected to be error free (estimated at 82% of total reads for 454 pyrosequencing; Huse et al. 2007), a cluster representing a single true allele should contain a single “dominant” allelic sequence consisting of the majority of reads in the cluster and a much smaller frequency of derived sequences that represent artifacts. A cluster containing more than one dominant sequence likely contains reads derived from more than one true allele, in which case the reads in that cluster are re-entered into the algorithm for further partitioning. Once the clustering rounds are complete, the final result of STC is a set of N clusters representing the N true allelic sequences for each sample.

The clustering algorithm does two things that help to solve the gray-zone and similar allele problems. First, clustering similar sequences together means that the more frequent artifacts are clustered together with the actual alleles from which they are derived, and thus artifacts will not necessarily be mistaken for alleles just because they are relatively common. Similarly, less frequent allelic sequences will end up forming their own distinct clusters with related artifactual sequences, even if the sizes of those clusters are relatively small. Second, by applying criteria to establish whether there is more than one dominant allele in a given cluster, true alleles with similar sequences can be differentiated from one another by refining the clustering until two legitimate clusters are formed. Clustering also has one additional advantage in that clusters that do not meet initial size criteria can be kept for cross-checking with known true alleles after clustering is complete. Such “small” clusters might represent artifacts – e.g. chimeras or divergent sequencing artifacts that appear early enough during PCR to generate many reads – or true alleles whose amplification efficiency is much lower than other alleles present in the original sample. If a given small cluster from one sample appears as a large cluster in multiple other samples, it can be assumed that the small cluster represents a true allele whose small cluster size was likely due to stochastic sampling effects. In essence, a strict size criteria can be applied during clustering to reduce the accumulation of false positives (small clusters that do not represent alleles), while cross-checking the resulting small clusters against the all samples can substantially reduce the number of false-negatives (true alleles not recognized as such).

## Methods and Materials

### Ethics Statement

This study was carried out in accordance with the protocol approved by the University of Texas Institutional Animal Care and Use Committee (permit # 07100201). Fish were collected using permit # SPR-0305-038 issued by the British Columbia Ministry of Environment.

### Data and Script Availability

Raw and processed data files and all scripts necessary for running the STC algorithm have been made available at the Dryad Digital Repository (http://dx.doi.org/10.5061/dryad.4fn4g). Users can use the provided scripts and data to generate the genotyping results described below, as well as applying them to their own data. Instructions for using the scripts are provided in the README file included in the repository.

### Sample Collection

We collected 364 threespine stickleback (*Gasterosteus aculeatus*) from four different populations on northern Vancouver Island (Table 1). Upon capture all fish were euthanized with a lethal dose of MS-222. Caudal fin clips were taken from each fish and stored in 90% ethanol for later DNA extraction. DNA was extracted from whole fin clips using Wizard Genomic DNA Purification Kits (Promega #A1120), following the protocols indicated by the manufacturer for animal tissue extraction. Initial DNA concentrations were obtained using Quant-iT PicoGreen kits (Invitrogen P11496) following manufacturer protocol.

**Table 1:** Population sampled and numbers of samples collected.

Pair	UTM coordinates	Samples amplified	Samples genotyped
Farewell Lake	314926E, 5564416N	114	90
Farewell Stream	314004E, 5564614N	50	44
Roberts Lake	318053E, 5566856N	133	105
Roberts Stream	316975E, 5567731N	67	56

UTM coordinates are zone 10U.

### PCR and Sequencing

It has been previously estimated that stickleback could have as many as six [59] and as few as two to four [36], [60] different MHC class IIβ loci. The publicly available reference genome assembly for stickleback [61] contains 5 annotated and 1 unannotated MHC class IIβ loci with associated ESTs [60]. The highly variable, polymorphic binding region in stickleback is located in exon 2 [59]. We designed forward and reverse primers that were conserved across these six MHC class IIβ loci, meaning these loci should amplify simultaneously [42], [62]. Our forward primer sequence (5'-TGTCTTTAACTCCACGGAGC-3') sits 32 base-pairs downstream from the start of exon 2, while our reverse primer sequence (5'-CTCTGACTCACCGGACTTAG-3') spans the boundary between exon 2 and intron 2. The amplicon generated by these primers is 213 base pairs long (253 base pairs including primers) and constitutes 70 (81 including primers) of the 92 amino acids of exon 2. Note that these primer sequences are very similar to those developed independently by Lenz et al. [42] for amplifying stickleback MHC class IIβ exon 2 sequences intended for reference strand-mediated conformation analyses (RSCA).

Each forward and reverse primer also contained a 15 bp barcode at the 5' end (Table S1). Each sample in our pyrosequencing run was initially amplified using a unique combination of forward and reverse barcodes so that reads associated with individual samples could be identified after sequencing. By using 20 (or more) barcodes on the forward and on the reverse primers we are able to multiplex up to 400 (or more) uniquely barcoded individuals into a single sequencing library. Our barcodes consisted of 10 base pair MID tags supplied by Roche for amplicon pyrosequencing, with an additional 5 base pairs from the beginning of another MID tag added on to the 3' end.

PCR reactions were performed in 50 ul total volume containing 25 ng of extracted DNA, 10 uL of 10X (-MgCl2) PCR buffer (Invitrogen), 300 µmol of MgCl2 10 µmol dNTPs, 20 µmol each of forward and reverse primers, and 1 unit of Platinum Taq DNA Polymerase (Invitrogen). The PCR program used for all samples was: initialize at 94°C for 120 seconds, 25 cycles of denature at 94°C (30 seconds), anneal at 57°C (30 seconds), and extension at 72°C (60 seconds), and a final elongation at 72°C for 240 seconds. Lenz and Becker [63] were able to substantially reduce the number of PCR chimeras when sequencing stickleback MHC class II using 25 PCR cycles and a 60 second extension, both of which we applied here. After PCR, samples were cleaned using Agencourt AMPure XP PCR purification (Beckman Coulter) according to the manufacturer's instructions, re-quantified using the Quant-iT PicoGreen kits used previously, and finally pooled into a single library at equimolar concentrations for sequencing. Samples were sequenced at the University of Texas Genome Sequencing and Analysis Facility on a Roche/454 FLX sequencer using titanium chemistry and standard amplicon pipeline procedures. The entire library was run on one-quarter of a picotiter plate.

### Stepwise Threshold Clustering

**Figure 1:**
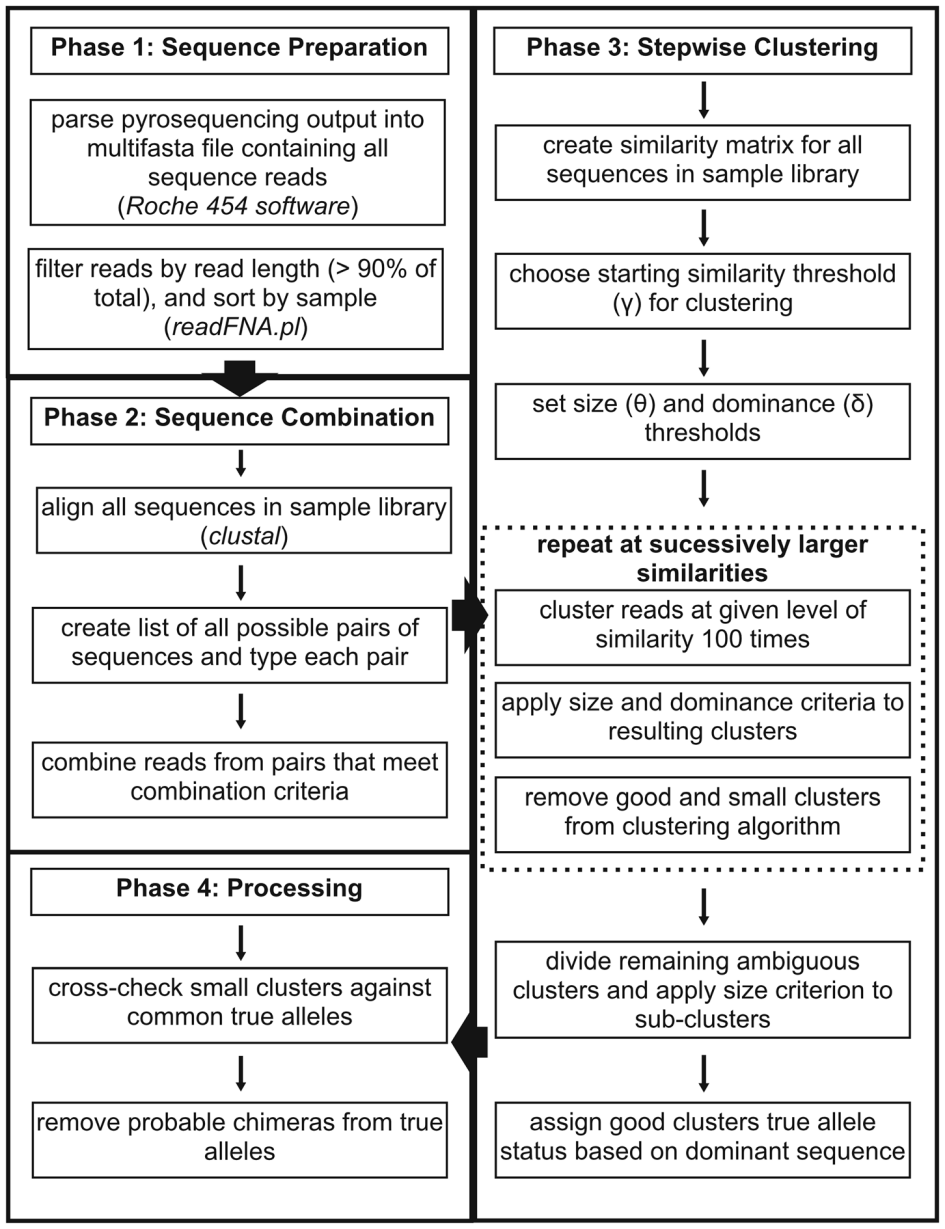
Outline of steps in STC genotyping. Programs and software used to implement each step are given in italics. The initial sff file was parsed using proprietary Roche software at the University of Texas Genome Sequencing and Analysis Facility. Filtering of reads and parsing samples by barcodes was accomplished using a custom perl script. Phases 2–4 were implemented in a custom R script. All scripts necessary for running STC have been uploaded to the Dryad Digital Repository (http://dx.doi.org/10.5061/dryad.4fn4g)

STC can be broken down into four phases: 1) sequence preparation, 2) sequence combination, 3) stepwise clustering, and 4) post-clustering processing (Fig. 1). Phase 1 consists of filtering reads and partitioning sequences among samples based on barcode sequences and can be performed using custom scripts or publicly available software [47]. Note that two phases of STC (2 and 3) are applied in succession to all individual sample libraries before moving on to phase 4. Commonly used terms and their description are provided in Table 2.

**Table 2:** Commonly used terms and definitions.

Term	Definition
sample	one individual to be sequenced (human, fish, mouse, bird, etc.)
sample library	all reads produced in a given sequence run for a single sample
run library	all reads produced by a single sequencing run (includes all samples)
read	individual, non-unique sequence produced during sequencing corresponding to the sequence of a single PCR amplicon
sequence	unique read produced during a sequencing run (many reads can correspond to the same sequence), and indicated by an # (e.g. #130)
true allele	sequence inferred to match an allelic sequence in the genome of a given sample
artifact	sequence inferred to not match an allelic sequence in the genome of a given sample
cluster	group of similar reads (or a single read) produced during phase 3 of STC
dominant sequence	sequence with most reads in a given cluster
subdominant sequence	sequence with second most reads in a given cluster
dropped allele	an allele originating from a small cluster in phase 3 that was made a true allele during phase 4
missing allele	an allele present in the genome of the sample does not appear as a true allele after STC is complete

### STC Phase 1: Sequence Preparation

The STC process starts with the raw read data in the form of a multiFASTA (.fna) file derived from the sff file generated by a single 454 sequencing run. We used a custom Perl script to parse this file (available at the Data Dryad Repository: http://dx.doi.org/10.5061/dryad.4fn4g). Each read corresponds to the sequence generated within a single well of pyrosequencing. This script identified the forward and reverse barcodes, and the amplicon sequence (hereafter, just sequence) for every individual read. Any read not containing a uniquely identifiable forward and reverse barcode was discarded. In addition, we applied a minimal read length filter, keeping all reads with intra-primer sequence of at least 200 base pairs (∼94% of the amplicon length). This was done to ensure that short reads derived from different sequences were not classified as the same sequence due to missing base pairs. Stricter criteria could be applied, such that only sequences within three base pairs of the expected amplicon length could be included. However, the efficiency of cluster classification is improved by including more reads. Once reads have been minimally filtered by length and organized by sample, and forward and reverse primer and barcode sequences removed, the reads are ready for phase 2.

### STC Phase 2: Sequence Combination

Previous approaches to genotyping typically flag sequences containing indels as artifactual and remove them from the analysis [44], [45], [53]. In STC we take a different approach. In phase 2, pairs of sequences are “combined” when one member of the pair has the correct number of basepairs (i.e. no open reading frames or stop codons) while the other member has an incorrect length but is otherwise identical or nearly identical to the first member. By combine, we mean that the reads associated with the second, artifactual member are converted to reads of the first, correct-length sequence.

The rationale behind this phase is straightforward: artifactual sequences contain important information about which sequences represent true alleles. This is especially true for reads obtained using 454 pyrosequencing, as the most common sequence errors generated during pyrosequencing are homopolymer insertion/deletion errors [56], which occur when the number of successive, identical base pairs in a sequence are over or under-called relative to the true sequence. By combining artifacts with very similar sequences from which they are clearly derived, we increase the weight of evidence that the more frequent sequence is a true allelic sequence. This tends to reduce ambiguity in the subsequent clustering phase, because true allelic sequences will be more dominant relative to other sequences in their cluster after being combined with clearly artifactual sequences.

To combine sequences we first divide all possible pairs of sequences in a sample library into five pair types: (I) pairs that differ by only an indel (II) pairs that differ by one insertion and one deletion (III) pairs differing by only one indel and one substitution (IV) pairs that differ by one indel and two substitutions (V) all other pairs, which are not combined. In types I, III and IV, the first member of the pair is always the sequence of the correct length. In type II, where the lengths are the same, the first member is always the more common sequence. We then apply three criteria to each pair to determine whether they should be combined. First, the first member of the pair must have the correct number of base pairs for the sequence of interest (e.g. 213 bp for our sequences). Second, the first member of the pair must be more common than the second member. Third, pairs can only be combined if the second, derived member is unique to that pair within that type. If, for example, one type III pairs contains sequences X and Z, and another type III pair contains Y and Z, then it is ambiguous whether Z is derived from Y or X and neither pair is combined. Finally, we note that, because every possible pair of sequences is evaluated before combining pairs, the length of the time for phase 2 increases with the square of the number of unique sequences present in the sample library.

### STC Phase 3: Clustering

The STC algorithm uses a variation on a formal Dirichlet process known colloquially as a Chinese restaurant table process. In the restaurant analogy, imagine 100 customers wish to enter a restaurant that can contain an infinite number of tables. The first customer enters the restaurant and sits at a table. The second customer enters and can choose to start a new table or to sit at the existing table. Every subsequent customer enters the restaurant and makes the same choice—sit at a new or existing table. Whether or not each new customer chooses to sit an existing table is directly proportional to how many customers are already at the table when the new customer enters. In a formal, discrete time restaurant table process objects (customers) start a new group (table) at some constant probability, or join an existing group (table) with a probability proportional to the size of each group (i.e. number of customers already seated at each table). The end result once all objects have been grouped is a set of groups that vary in the number of objects they contain.

Our clustering process uses a similar mechanism to form clusters by sequentially taking each read (customer) in a sample library and either using it to start a new cluster (table) or allowing it to join an existing cluster (table). The process is quasi-Dirichlet because the reads (customers) are not clustered using probability rules based on cluster size. Rather, reads are added to clusters based on sequence similarity criteria. Specifically, at the point at which a given focal read is introduced, each existing cluster is assigned the sequence of the most frequent read contained within said cluster. For example, if cluster A contains two reads corresponding to sequence X and one corresponding to sequence Y, the cluster takes on the identity of sequence X (i.e. cluster A is assigned the sequence of X). The given focal read then either added to the most similar existing cluster, assuming the similarity between the focal read and the cluster is above predefined similarity threshold (γ, see Table 3 for list of parameters set by the user) or it starts a new cluster. Note that once a read has been placed in a cluster, any reads sharing the same sequence will automatically be placed in that cluster when they are introduced into the process, meaning reads with the same sequence will never end up in different clusters. We note that this process is very similar to a process independently developed by Prosperi et al. [64] to delineate HIV and Hepatitis-C viral sequences into different subtypes.

**Table 3:** User-defined parameters for STC.

Parameter	Name	Definition	Recommended starting value	Value used
γ	similarity threshold	minimum similarity required between focal read and cluster for read to join cluster, increases in successive clustering rounds	start at minimum similarity between two reads in the given sample library (e.g. 60%)	Varies between samples
θ	size threshold	minimum ratio of reads in a cluster to reads in a sample library necessary for the cluster to be classified as “good”	1/maximum expected number of alleles/2	1/22
δ	dominance threshold	minimum ratio of dominant to subdominant reads necessary for the cluster to not be classified as “ambiguous”	4∶1	4.55∶1
ε	common allele	an allele must be present in at least this many samples to be considered common for the purposes of cross-checking in phase 4	3	3

Recommended starting values are offered because the optimal values will vary both by data set and the degree to which the user wishes to balance false positives and false negatives. Values used for the data set presented here are given in “value used” column.

STC is designed to identify clusters representing alleles that are very dissimilar from other alleles before gradually breaking apart clusters representing very similar alleles. To accomplish this goal, the sequence similarity threshold (γ) for joining existing clusters is gradually increased over successive rounds of clustering. At the beginning of phase 3, γ takes the similarity between the most dissimilar reads for a given sample and increases by a set amount (e.g. 1%) during each successive round of clustering. This means that each successive read is more likely to form a new cluster in later rounds than in earlier ones. Clustering can, in theory, continue to γ = 100%. In practice it is much better to end slightly below this level (γ = 97%) in order to avoid separating artifactual sequences with minor errors from the clusters to which they belong.

Note that whether a focal read joins an existing cluster or starts a new cluster is entirely deterministic and depends only on the predefined sequence similarity threshold (which differentiates STC from a formal Dirichlet processes). However, during each round, each focal read is chosen at random from the remaining pool of reads until all reads are clustered, introducing a small amount of stochasticity into the final cluster configuration (sometimes two or three different configurations can occur depending on the order of clustering and value of γ). To account for variation introduced by random ordering of reads, we repeat the clustering 100 times during each round, starting with a randomly chosen sequence each time. The most common cluster assignment (the mode) for each read among the 100 replicates is used to determine to which cluster each read is assigned in each round.

At the end of each round, every cluster assumes the sequence identity of its most frequent read and is assigned to one of three categories – good, small, or ambiguous – based on the two criteria. The first, which we refer to as the size criterion, states that a cluster must contain a certain proportion, θ, of the sequence reads from the focal sample library. The second, the dominance criterion, states that the frequency of the most common (dominant) read in a cluster divided by the frequency of second most common read (subdominant) must be greater than the threshold ratio δ. Both θ and δ are set before clustering begins and do not change between rounds of clustering. Clusters that meet both criteria after each round are classified as good clusters and all reads contained therein are exempt from further rounds of clustering. Clusters that meet the dominance criterion but not the size criteria are considered small clusters and are also considered exempt. Clusters that do not meet the dominance criterion (e.g., contain two or more abundant sequences), potentially contain more than one true allele. These clusters are classified as ambiguous and are retained for the next round of clustering at a more stringent threshold whether or not they meet the size criteria.

Although the thresholds associated with the two criteria must be set heuristically by the user, they can be adjusted to better trade-off false positives and false negatives. Setting θ too low means some small clusters that actually derive from artifactual sequences may be categorized as good (an increase in false positives). Alternatively, setting θ too high will result in an increase in false negatives, although this increase can be mostly be offset during phase 4, meaning its better to set θ somewhat conservatively. For example, given no stochastic or amplification bias effects, the lowest proportional size of each cluster will simply be one divided by the maximum expected number of alleles (N_max_, assuming no homozygotes). Because there will inevitably be some clusters smaller than that the 1/N_max_ ratio due to amplification bias or stochastic sampling of reads from the library, an appropriate starting value for θ would be to divide 1/N_max_ again by some constant C to reduce the minimum size a bit further. For example, with 6 different MHC loci, N_max_ = 12. An appropriate starting value for θ would be 1 divided by 12 divided by again 2 or 1/24. In this instance, the size criterion states that a cluster must contain at least 1/24 of the total reads for that sample. An appropriate starting point for δ is to consider that experiments have shown that about 18% of reads in a given run represent artifacts [56]. This means that the ratio of the dominant sequence to the subdominant in a cluster will likely be on average no more than 0.82/0.18 = 4.55, and usually much greater, so δ = 4.55 makes a good starting value.

Finally, after clustering is complete, it is possible that some clusters will remain classified as ambiguous. Either these clusters represent two very similar alleles, one allele with an additional frequent artefact, or zero true alleles. These clusters are dealt with as follows. Each ambiguous cluster is divided into two sub-clusters whose size (number of reads) is proportional to the relative frequencies of the two most frequent reads (dominant and subdominant) in the cluster. These two sub-clusters are then checked against the size criterion. If a sub-cluster passes the size criteria, then it is considered a good cluster. Otherwise the sub-cluster is classified as a small cluster. Additionally, it is sometimes the case that one of the top two sequences in a cluster will not be the correct length, in which case the third most frequent sequence is treated as the subdominant sequence and the same rules are applied.

### STC Step 4: Post-clustering processing

Phase 4 consists of two stages: cross-checking all small clusters against good clusters and checking allele sequences for possible chimeras. As noted by Sommer et al. [53], some alleles may tend to not be genotyped due to low amplification efficiencies, or alleles may be missing from sample libraries simply due to chance (we refer to such cases as “missing” alleles). In other cases alleles present in the genome of the sample will be present in the sample library but won't produce enough reads to pass the size criterion (we refer to such cases as “dropped” alleles). To check for dropped alleles, small clusters are cross-checked against commonly occurring good clusters across all samples. Good clusters are classified as common if they occur in at least ε samples after phase 3 (e.g. at least three other samples). If the identity of a given small cluster matches a common good cluster, then it is assumed that the cluster “dropped out” due to stochastic effects and can be included in the list of good clusters for that particular sample.

Cross-checking small clusters in this way also has an added benefit, because the frequency at which a cluster drops out during phase 3 provides information as to whether the allele likely amplifies with low efficiency. Because such alleles will tend to drop out more frequently than other alleles for a given value of θ, cross-checking clusters also allows the user flexibility in adjusting the value of θ to control the rate of false positives and false negatives. Users can be relatively conservative with assigning the value of θ, knowing that dropped clusters (i.e. potential false-negatives) can be cross-checked and included in the final good cluster at the end. Once small clusters have been cross-checked against common good clusters, all good and dropped clusters for each sample are officially assigned true allele status, whereby the dominant sequence in each cluster is inferred to represent a true allelic sequence.

Lastly, true alleles are checked to see if they are likely to be PCR chimeras. Chimeric sequences can occur during the initial PCR stage when incompletely extended primer sequences subsequently anneal to a different template in a later cycle, or by template switching during extension. In either case, the chimeric daughter strand will resemble one parent sequence over one portion of its length, and a different parent sequence over the other portion. If the number of PCR cycles has been kept to a minimum during read generation [63], chimeras are unlikely to be present in large numbers because heteroduplexes are more likely to form during the later stages of PCR [65]. Nonetheless, it is recommended that any alleles obtained for a given sample library be checked for possible chimeras. A number of common methods for detecting chimeric sequences have been published [66]–[68], although these were designed for large 16S rRNA data sets where the number of potential parent templates and sequences are large and unknown. In the context of MHC genotyping, alleles can be checked for chimeras using visual inspection of alignments [45], or with custom scripts that check alleles against all sequences present in a given sample library [53]. In STC, each true allele is scanned to see if it looks like a close recombinant (daughter sequence) of two other true alleles (parents) in the same sample. For each possible recombinant allele, all the samples containing that allele are scanned for the possible parent alleles. In cases where the recombinant alleles occurred with possible parent alleles in all cases, we classified the recombinant as a chimera and removed it from the final data set. Recombinant alleles that do not co-occur with possible parent sequences in all samples are assumed to have resulted from natural recombination/gene conversion events between loci and are not classified as chimeras.

### Amplification Efficiency

To check whether variation in the rate at which alleles were dropped during phase 3 was potentially related to amplification efficiency, we estimated the correlation between average relative cluster size (proportion of sample library reads in a cluster) and the rate of dropping (fraction of total occurrences where an allele was initially dropped in phase 3). For each allele, we estimated the relative cluster size both overall and only in samples where an allele was not dropped. Alleles that are amplified at lower efficiencies should have both smaller relative cluster sizes within sample libraries (even in cases where those alleles are not dropped) and elevated probabilities of dropping compared to other alleles. We therefore expected a negative correlation between cluster size and rate of dropping if low amplification efficiency was causing some alleles to drop out more than would be expected by due to stochastic effects alone.

### Repeatability of STC genotyping

Duplicate PCR reactions were run for 21 total samples. When both duplicates achieved the minimum sample library size, we compared the STC output for each duplicate to test the consistency of STC across different libraries for the same samples. We also cloned and sequenced four samples for verification of our genotyping method. We used the same PCR conditions used to amplify samples for pyrosequencing. The PCR products were purified using QIAquick PCR Purification Kits (Qiagen 28014) and cloned into a vectors using a pCR 2.1-TOPO TA kit provided by Life Technologies (K450001). After overnight growth, individual clones were amplified using M13 forward and reverse primers. Amplified clone sequences were purified using the same QIAquick kits and sequenced directly on an Applied Biosystems AB 3730 sequencer at the University of Texas ICMB DNA core facility. We originally targeted 100 clones per sample, but found that only 50–60 clones were needed to verify the most diverse sample.

### Relationship between read number and allele number

Samples that yield fewer sequence reads than typical are prone to having alleles with zero corresponding sequence reads in their sample library (i.e. missing alleles) due to stochastic under-sampling of reads during sequencing. Moreover, the probability of such missing alleles will be magnified when those alleles also amplify will low efficiency. As a result, allelic diversities may be underestimated for some samples with small library sizes. To test for this possible bias, we estimated the correlation between library size (read number) and allele number within each of the four populations of stickleback. In any populations where the correlation was significant, we re-estimated the correlation using increasingly larger minimal samples sizes (up to 800 reads), to ask at what point the correlation was negligible or became statistically insignificant (keeping in mind that the power to detect a significant correlation will decrease as more samples are removed from the analysis due to an increased minimal sample size).

### Visualization of allele and cluster similarity

In order to help visualize the clustering process for our individual example sample library, we created a two dimensional plot of all the sequences present in that sample library using multidimensional scaling (MDS). Briefly, MDS attempts to project the N-dimensional distances between objects (i.e. sequences) into a two dimensional space. Sequences placed closer together in the space are more similar to each other than sequences placed further away. One advantage of using MDS to view sequence relationships is that it can be based on the same similarity matrix used by STC to cluster sequences, and thus provides a visual representation (though not complete replication) of the STC process. We used the *pcoa* function in the R package *ape* [69] to generate principal coordinate axes of genetic similarity based on the percentage of shared base pairs. The first two MDS axes can be used to create a plot of genetic similarity. In addition to the MDS plot, we also created a neighbor-joining tree of the most common sequences across all samples to visual sequence similarity among alleles. We used the *bionj* function in the *ape* package, which implements the method of Gascuel [70] for producing neighbor joining trees, and the *plot.phylo* function for visualizing the tree. All visualizations were done using the R statistical programming language, version 2.15.1 [71].

### Statistical comparison between populations

We used the results produced by the STC algorithm to make three different comparisons between our four populations. First, we used an ANOVA to determine whether our populations differed in the mean number of alleles per fish. Habitat (lake or stream), population pair (Roberts or Farewell), and their interaction were used as factors in the ANOVA. Second, it has been hypothesized that divergent selection among habitats (due to contrasting parasite communities) could lead to the divergence in MHC genotypes. We therefore tested whether our lake and stream populations differed significantly in overall MHC allele composition using the GLM-based approach advocated by Warton et al. [72] and implemented in the R package mvabund [73]. This approach first uses separate GLMs to estimate the effects of predictor variables (i.e. habitat and pair) on the probability of having each MHC allele. Individual test statistics (e.g. likelihood ratios or wald statistics) from each GLM are then added together to create an overall test statistic for each predictor, and permutations of the original data are followed by recalculation of the overall test statistics to produce p-values. This approach has an additional advantage of controlling for correlations in the response variables among individuals, as might occur due to linkage disequilibrium among loci. Warton et al. [72] have shown their approach to be both more powerful statistically and much less prone to confounding dispersion and location effects than are similar approaches such as ANOSIM [74] or PERMANOVA [75]. Our p-values were calculated using 1000 permutations. Lastly, we used log-likelihood ratio tests (G-tests) to test whether the presence or absence of each allele was significantly associated with habitat within pairs. We restricted this analyses to testing differences between lakes and streams within pairs. P-values were corrected for multiple comparisons by applying a false discovery rate of 5% [76]. All statistical analyses were implemented using the R statistical programming language, version 2.15.1 [71].

The most recently proposed method for using NGS to genotype MHC loci requires every sample to be run in duplicate [53], thus decreasing by half the number of samples that can be genotyped in a given run. We wished to determine the degree to which genotyping half as many samples per population would effect the probability of finding significant differences between habitats in individual allele frequencies using the analysis described above. We randomly sub-sampled half the samples from each population 1000 times without replacement and recalculated p-values for each subsample. We then calculated the percentage of the 1000 subsamples in which each allele was found to be statistically significantly different between habitats (after accounting for multiple comparisons within each subsample). We would expect that, with our increased sample size, we would be much more likely to identify alleles that differ significantly in frequency between habitats than if we had genotyped only half as many samples.

## Results

### Illustration of STC using one sample

#### Phase 1

A single stickleback sample (hereafter, sample X) from the Roberts Lake population (sample ID 490 in Table S2) was chosen to illustrate the STC process in detail. Sample X had, after initial quality and length filtering, 330 reads in its sample library. These reads corresponded to 101 unique sequences, including 72 sequences that only appear once in the sample library (Table S3 contains a summary of all 101 unique sequences associated with sample X). This sample was chosen partly because its 6 true alleles and 330 reads fell near the median of both distributions. More importantly, this sample illustrates clearly three of the processes unique to STC: 1) the gradual separation of clusters representing alleles as the similarity threshold γ is increased, 2) the cross-checking of small clusters against common alleles to reduce false negatives, and 3) the delineation of two true alleles that differ by fewer than 3 base pairs.

#### Phase 2

In this section, unique sequences will be referred to by the including a # at the beginning of the numerical sequence ID (i.e. #1234). Clusters of sequences (good, small, or ambiguous) are designated by an X at the beginning of a numerical sequence ID (i.e. X1234), where the ID refers to the dominant sequence in the cluster.

Of the 101 unique sequences in the sample library, 59 were of the correct length (213 base pairs). Thirty-three sequences were off by a single base pair (212 or 214 base pairs), 7 by two base pairs, and 2 by three or more base pairs (Table S4). After aligning and checking all 3160 unique pairs of sequences for indel and substitution differences, we found 21 type I pairs (i.e. sequences differing by a single indel). One sequence (#26826) differed from two sequences by one indel and was combined with the more common of the two. Combining these remaining 20 type I pairs of sequences left 81 unique sequences in the sample library. We found 4 type II and 27 type III pairs which also met our criteria for combining pairs. This resulted in a further decrease to 67 unique sequences, while the total number of sequence reads remained at 330. A summary of all pairs combined during phase 2 for sample X is contained in table S4.

#### Phase 3

**Figure 2:**
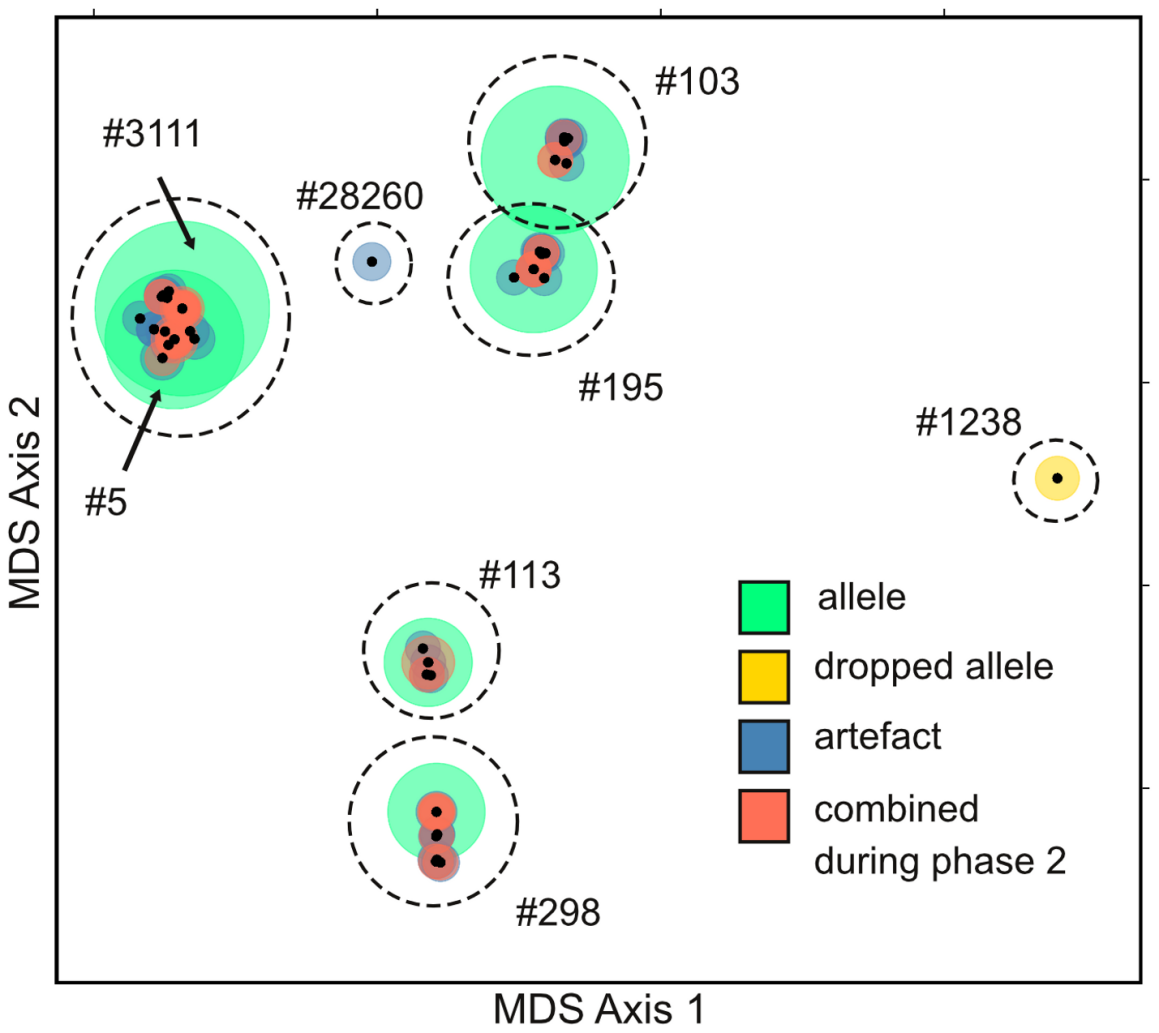
MDS plot of sequences in sample library X. Small black dots indicate the 101unique sequences present in the sample library. Sequences have been plotted on the first two MDS axes generated using the same similarity matrix used during clustering, such that more similar sequences are closer together. The color of the larger circles indicates the final status of each sequence, whereas the size of each circle is proportional to the number of reads in the sample library that match that sequence (see Table S3 for list of sequences and their respective read numbers). Sequences indicated in red were combined with other more frequent sequences during phase 2. Sequences indicated in blue were deemed to be artifactual sequences. Allele #1238 (yellow) was not considered a good cluster in phase 3 (too small) but was considered a true allele after cross-checking in phase 4. The green circles indicate sequences corresponding to the other 6 true alleles. The dotted lines indicate the seven clusters (4 good, 1 ambiguous, 2 small) after the 97% similarity threshold had been reached in phase 3.

We set the size threshold for accepting clusters as good clusters at θ = 1/22 = 0.045 and the dominant to subdominant ratio threshold at δ = 4/1. Note that these thresholds are only slightly different than the baseline thresholds suggested in the methods and were determined heuristically by re-running STC on a subset of samples to minimize false positives. The minimum sequence similarity among all pairs of sequences in the sample library was 60%, so we began clustering at γ = 60%. Table 4 contains a summary of clusters generated during each round of clustering for sample X (described below). Figure 2 visualizes the clusters in two-dimensional similarity space.

**Table 4:** Clustering round results for example sample library.

Rounds	minimum similarity % (γ)	good	small	ambiguous	reads left	sequences left	good clusters added	small clusters added
1–10	60–69	0	0	1	330	67	-	-
11–22	70–81	0	1	1	326	66	-	#1238
23–26	82–85	0	1	2	326	66	-	-
27–28	86–87	0	1	3	326	66	-	-
39–30	88–89	0	2	3	323	65	-	#28260
31–37	90–96	2	2	2	252	52	#113, #298	-
38	97	4	2	1	140	23	#103, #195	-

Good and small cluster numbers are cumulative. Ambiguous clusters indicates the number of ambiguous clusters created during each round that carried over to the next round. Clustering rounds where the results did not change are grouped together. Reads and sequences are left after (not before) clustering in a given round.

As expected, clustering all 330 reads at γ = 60% resulted in a single giant cluster. The dominant and subdominant sequences, #3111 and #103, were represented by 73 and 42 reads respectively. This single cluster clearly does not meet the dominance criterion (73/42 = 1.74, which is smaller than δ = 4). The same result was achieved when clustering their reads through γ = 69%. At γ = 70% sequence similarity, two clusters were formed. One cluster (dominant sequence #1238) consisted of a single sequence of 4 reads, which classified it as a small cluster (4/330 = 0.012, which is less than θ = 0.045). The other 326 reads were placed in a single large cluster whose dominant and subdominant sequences were again #311 and #103. This single ambiguous cluster was produced at similarity thresholds through γ = 81%.

At γ = 82%, two clusters were produced. The #3111/#103 cluster remained ambiguous as before. A second cluster with dominant and subdominant sequences #298 and #113 was also formed. However, this additional cluster did not meet the dominance threshold (30/27<4/1), and was also classified as ambiguous. At γ = 85% the cluster dominated by #3111 split into two clusters, with dominant/subdominant sequences of #3111/#5 and #103/#195 respectively. However, none of the three clusters passed the dominance criterion. At γ = 88%, an additional cluster consisting of 3 reads of a single sequence (#28260) was formed and classified as a small cluster (3/330<0.045).

At γ = 90%, four total clusters were formed. The aforementioned two clusters #3111/#5 and #103/#195 remained ambiguous. One of the other clusters, the cluster with the dominant sequence #298 met the size (41/330>0.045) and dominance (30/3>4) criteria and was classified as a good cluster. The cluster dominated by sequence #113 also met both criteria (31/330>0.045 and 27/1>4) and was also classified as a good cluster. At the final round of γ = 97%, one of the previous ambiguous clusters (#103/#195) split into two distinct clusters, both of which passed the criteria and were considered good clusters. At this point there were 4 good clusters corresponding to sequences #298, #113, #103 and #195, two small clusters corresponding to sequences #1238 and #26820, and one remaining ambiguous cluster with dominant and subdominant sequences #3111 and #5 that differed by only two base pairs (Fig. 2).

To determine whether our remaining ambiguous cluster should be considered two separate clusters, we divided the total number of reads in the cluster (140) between the two dominant sequences in proportion to the number of reads for each sequence. The top two sequences accounted for 111 reads, of which sequence #3111 accounted for 73 reads (65.8%) and sequence #5 accounted for 38 reads (34.2%). Thus, the two hypothetical sub-clusters were assigned 65.8% and 34.2% of the total cluster reads, giving them 92 and 48 reads respectively. In this case, both sub-clusters had greater than 4.5% of the total reads in the sample library (335) and were classified as good clusters. Thus at the end of the phase 3 we were left with 6 good clusters representing sequences (X298, X113, X103, X195, X311, and X5), and two small clusters consisting of one rare sequence each (X1238 and X28260).

#### Phase 4

To cross-check our small clusters against common good clusters, we set ε = 3, which means that a sequence would have to be the dominant sequence of a good cluster in at least three other samples to be considered common. Of the two small clusters carried over from phase 3 in our example, good clusters containing #1238 were present in 3 other genotyped samples, whereas #28260 was not classified as a good cluster in any other sample. Therefore, we added the cluster representing sequence #1238 to the list of good clusters for sample X. The cluster containing #28260 was considered an artifact. At this point, we inferred that the dominant sequences in our seven good clusters represented true alleles originally present in sample X. None of the seven true alleles was classified as a chimera (table 5).

**Table 5:** Alleles with recombinant sequences.

allele	total occurrences among all samples	occurrences with possible parent sequences	% of occurrences with parents	Classified as chimera?
#1176	1	1	100	yes
#1487	1	1	100	yes
#1692	1	1	100	yes
#2720	1	1	100	yes
#3584	1	1	100	yes
#5875	1	1	100	yes
#375	2	1	50	no
#1337	4	4	100	yes
#562	4	2	50	no
#512	14	4	29	no
#1238	14	1	7	no
#182	15	7	47	no
#175	17	2	12	no
#262	24	1	4	no
#1	34	28	82	no
#195	38	15	39	no
#717	47	1	2	no
#103	75	2	3	no

### Applying STC to genotype stickleback from four populations

We obtained 206,453 sequence reads from one quarter of a complete pyrosequencing run. The raw sff file has been deposited at the NCBI sequence read archive (accession number SRR1177032). After removing reads that had intra-primer sequences of less than 200 base pairs, we were left with 156,841 reads (76% of total reads). Of those, 136,861 (66% of total reads) could be assigned to a specific sample based on barcode sequences. Of the initial 385 individual PCR reactions (364 samples plus 21 duplicates), 359 had at least one read associated with them after initial filtering (Table S2). We set an initial cutoff of 80 reads per sample for subsequent genotyping using STC, leaving us with 301 total samples (295 samples plus 6 duplicates) to be genotyped. The mean number of reads per genotyped sample was 442 (median: 318, range: 81–4687). Of the 301 samples to be genotyped, 20 had more than 1000 reads associated with them. We elected to randomly subsample without replacement 1000 reads from each of these 20 sample libraries for genotyping. This substantially reduced the total run time of the STC algorithm (which scales exponentially rather than linearly with read number). After applying the minimum cutoff of 80 reads and sub-sampling to 1000 reads, the mean number of reads per sample was 380.

After STC was complete, we had identified 244 unique true alleles, of which 101 were present in only one sample (“singletons”, Table S5). We expected relatively few chimeras to be recognized as alleles because of the precautions taken during PCR and because chimeric sequences would tend to generate small clusters in samples where they occurred. We identified 18 potential chimeric (or naturally recombinant) alleles (Table 5). Seven of these alleles (six singletons) were present with potential parent sequences in 100% of the samples where they were identified as alleles. We removed these seven probable chimeric alleles from the final data set. Potential parent sequences were found in 50% or less of the samples in which the other 11 recombinant alleles were identified, suggesting these 11 alleles are likely naturally segregating recombinants. Removing chimeric alleles left us with 237 true alleles overall, including 96 singletons.

Of the remaining true alleles, 218 were the correct length of 213 base pairs (Table S5). The remaining 19 true alleles were only 212 basepairs long (15 singletons). Many of these 19 may be true false positives, while others could be naturally segregating variants, including one allele that appeared in eight different samples (allele #388, Table S5). Note that all of these 212 bp alleles had average cluster sizes greater than 0.065 – we used a cutoff of θ = 0.045 – indicating that a more stringent size criterion for clusters would still include many of these true alleles with incorrect lengths. However, we conservatively removed all of these 19 true alleles from our final data set before performing our statistical analysis, leaving 218 true alleles including 81 singleton alleles. All true allele sequences present in at least 5 samples and at least 213 base pairs long have been deposited in the NCBI GenBank (accession numbers KJ782461 – KJ782548).

**Figure 3:**
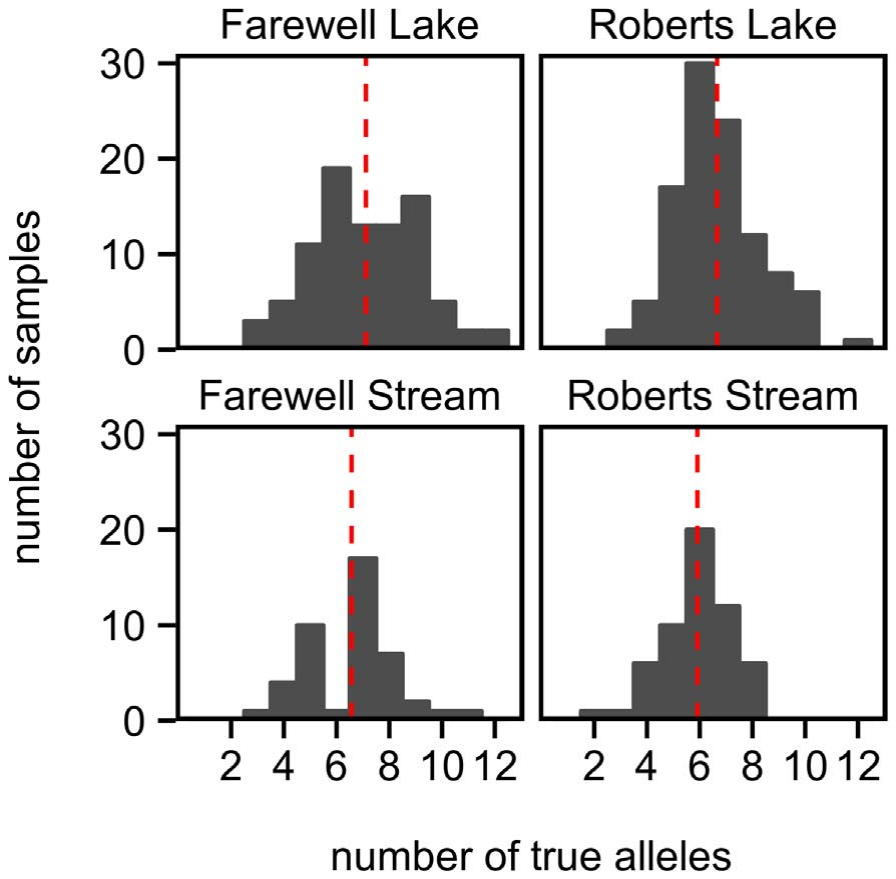
Number of alleles genotyped per sample. The red dashed lines indicate the mean number of alleles per sample for each population.

The overall average number of alleles per sample was 6.62 (mode: 6), although population averages ranged from 5.9 to 7.1 (Fig. 3). This is about 1–2 more alleles on average than have been found in previous studies of stickleback MHC using conformation based methods [62], [77], [78]. However, significantly higher diversities have previously been found using NGS compared to conformation based approaches in Scarlet Rosefinches (*Carpodacus erythrinus*) [49]. Our results, like theirs, are likely due to the increased sensitivity of NGS, which is more likely to detect alleles that amplify at lower efficiencies [53], [63].

### Dropped alleles and amplification efficiency

After phase 3 we had identified 9561 total small clusters among all of our samples, most of which were inferred to be sequencing artifacts. However, of those 9561 small clusters, 229 were classified as true (i.e. dropped) alleles after cross-checking against common good clusters during phase 4. The average number of such dropped alleles was 0.76 per sample (range: 0–4). Of the 301 genotyped samples, 164 (54%) had at least one dropped allele. Not all alleles were equally likely to be dropped. Of the 218 true alleles, 170 were never dropped (Table S5). Of the remaining 48 alleles, the average percentage of samples where a given allele was dropped was 18%. The least frequently dropped allele was dropped in only 2.6% of the samples in which it occurred, whereas the most frequently dropped allele (#670) was dropped in 73% of the samples where it occurred. A number of other alleles were also dropped at relatively high frequencies (>40%, Table S5), suggesting that they may be amplifying at relatively low efficiencies with our primer pair. None the less, given the diversity of MHC alleles overall, it is probably inevitable that some alleles will be amplified with relatively lower efficiency. More importantly, this result highlights the value of STC in diagnosing which alleles may be subject to amplification bias when genotyping MHC loci.

**Figure 4:**
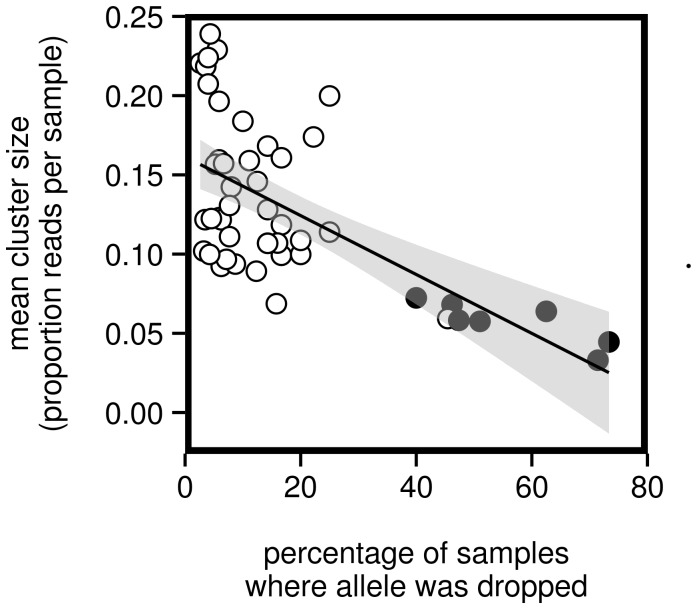
Negative correlation between average cluster size and frequency of dropping. Each point indicates a single allele. Only alleles that were dropped in at least one sample are plotted (n = 43). Cluster size is calculated as the average of the proportion of reads represented by the allele among all samples containing the allele. The solid line indicates the best-fit linear regression. The 95% confidence band for the regression is indicated in gray. Alleles associated with the “divergent” allele cluster (see Figure S2) are filled in black.

Considering only the 48 alleles that were dropped at least once, we found a significant negative correlation between the percentage of samples in which the allele was dropped and the average cluster size for alleles (r = −0.64, P = 1×10^−7^, Fig. 4). This correlation remained strong and significant if we calculated the average cluster size using only good clusters (r = −0.44, P = 0.001, Fig. S1). However, the correlation seemed due in large part to a small group of samples that had both small cluster sizes and a greater propensity to be dropped than other samples (filled circles in Fig. 4). We were curious about whether such potential low-efficiency alleles shared any sequence similarity. Of the eight true alleles dropped in at least 40% of samples where they occurred, seven were placed together in a clade of sequences that were distinctly divergent from other alleles (Fig. S2). The average pairwise similarity between members of this group to all other alleles was 65%. By comparison, alleles within this divergent group shared on average 99% sequence identity (∼2 basepairs difference), whereas alleles not in the divergent group were on average 87% similar. One possibility is that this group of alleles (which includes the above mentioned seven alleles plus two more) derive exclusively from a single MHC locus with slightly different primer sequences than the ones used here. Interestingly, 250 of 302 genotyped samples had at least one of the nine divergent alleles, and no sample had more than two, lending some support to the hypothesis that the alleles originate from a single locus that amplifies with low PCR amplification efficiency.

### Cloning

We sequenced bacterial clones from a total of four different samples (cloning results are summarized in Table 6). We initially targeted 100 clones per sample. In the case of two samples, we matched sequences to all alleles identified during STC after 5 and 34 clones respectively, although we continued sequencing up to 53 and 57 clones to catch any additional rare alleles potentially missed by STC. We found perfect congruence between the sequences identified by cloning and by STC in these two samples. We managed to isolate and sequence only 5 and 9 clones for the latter two samples due to bacterial contamination. For both of the partially cloned samples, all the clones exactly matched alleles found by STC, except in one case where a clone was clearly a chimeric recombinant of two other sequences present in the sample. However, due to the limited number of clones sequenced, we were only able to match 4 of 6 and 5 of 11 alleles identified by STC in those two samples. Our cloning was able to identify a singleton allele present in only one sample in our run (allele #7059 in sample ID 1368), suggesting that, in principal, singleton alleles identified by STC are not necessarily artifacts of the sequencing process. Overall, none of the cloning results contradict any of the results from STC genotyping, albeit in a limited number of samples.

**Table 6:** Cloning Results.

sample ID	good clusters	dropped alleles	total alleles	number of clones	total matches	alleles matched	mismatched clones
383	2	0	2	53	53	2	0
468	6	1	7	57	57	7	0
1024	11	0	11	9	9	5	0
1368	6	1	7	5	4	4	1

A mismatched clone sequence is a sequence that did not exactly match one of the alleles identified by STC. The one mismatched clone in sample 1368 was a chimeric sequence of two other alleles.

### Duplicated Samples

We originally amplified 21 different samples in different PCR reactions in order to compare the repeatability of the STC algorithm on the same samples (hereafter duplicates are referred to as “B” samples, Table S2). The results from our duplicated samples are summarized in table 7. Of those 21 samples, only 6 generated enough reads (>79) in both the A and B samples to be genotyped in duplicate. STC produced identical genotypes in the A and B samples in 2 of the 6 duplicate pairs. In 3 of the remaining 4 pairs, the B sample was missing allele #83 or #655, two of the most frequently dropped (and divergent) alleles mentioned previously. In one case (sample ID 403), the B sample had one read representing the missing allele #83, but the cluster was not large enough to be good or to be added during phase 4 (i.e. at least 3 reads in total). In the last duplicated sample (sample ID388), all three dropped alleles in the A sample (#83, #162, and #103) were not present in the B sample, suggesting that low efficiency accounts for their disappearance from the B sample as well. Overall, STC consistently identified the same true alleles in duplicate samples, with the exception of alleles that were identified (by STC) as alleles that tend to be dropped at high frequency. Additionally, it was not always the duplicate with more reads in which more alleles were identified, although alleles were generally not missing from duplicates that had at least 200 reads (Table 7). This implies (not surprisingly) that increasing the read number helps to reduce the likelihood of missing alleles that amplify at low efficiency. In fact, our duplicate results suggest that the frequently dropped alleles may, in fact, be missing (i.e. not be genotyped despite being present in the genome) at fairly high rates in samples with a small number of reads.

**Table 7:** Genotyping results from duplicated (A and B) samples.

sample ID	# reads (A)	# reads (B)	# of alleles (A)	# of alleles (B)	# of alleles shared	same alleles?	missing allele(s)
1385	270	143	7	7	7	yes	-
1397	765	114	7	7	7	yes	-
403	127	196	9	8	8	no	#83
82	400	440	7	6	6	no	#655
1361	285	149	5	4	4	no	#83
388	1000	109	6	3	3	no	#83, #130, #162
1076	158	176	7	8	1	no	-

### Relationship between library size and allele number

**Figure 5:**
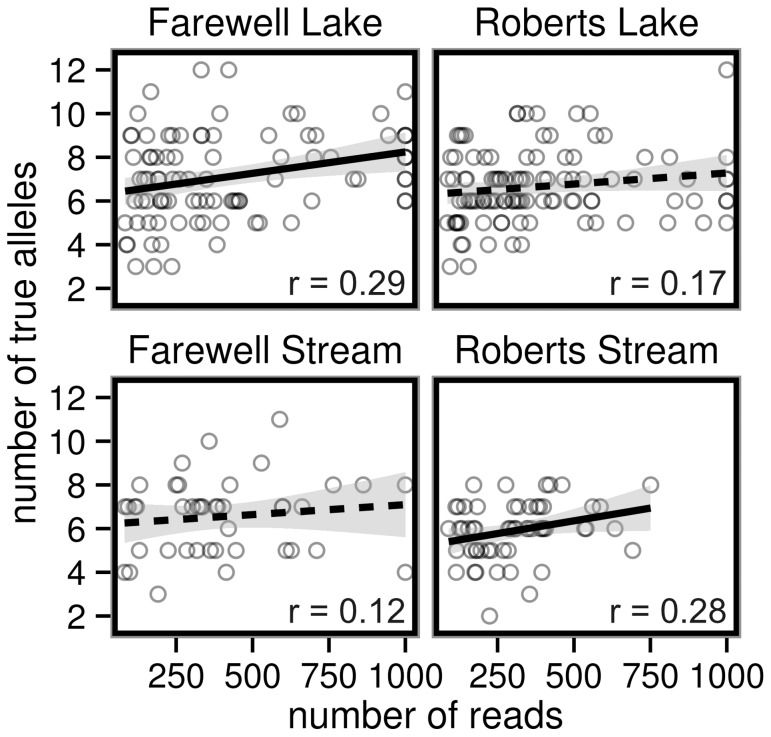
Correlations between allele number and library size. Each point indicates a genotyped sample. Samples with more than 1000 reads were sub-sampled to 1000 reads. No B (duplicate) samples were included to avoid pseudo-replication. The lines indicate the best-fit linear regressions for each population. Solid lines indicate correlations that are statistically significant at α = 0.05. Dashed lines indicate correlations that are not statistically significant. The 95% confidence bands for each regression are indicated in gray.

The correlation between sample library size (number of reads) and allele number ranged from r = 0.12 to r = 0.29 in the four populations (Fig. 5). The correlations were significantly different from zero in two of our four sampled populations, and marginally so in another (Fig. 5), indicating that the estimated number of true alleles generally increased with sample library size. Specifically, the expected number of true alleles at 80 reads and at 1000 reads increased by 0.8 alleles at the lowest (Farewell Stream) and by 2.1 alleles at the highest (Roberts Stream). Recalculating the correlations after removing dropped alleles from the analysis resulted in non-significant correlations that are closer to zero for all four populations (Fig. S3). Overall, these results suggest there may have been some bias introduced by variation in read number with samples tending to miss alleles up to a certain minimum library size.

**Figure 6:**
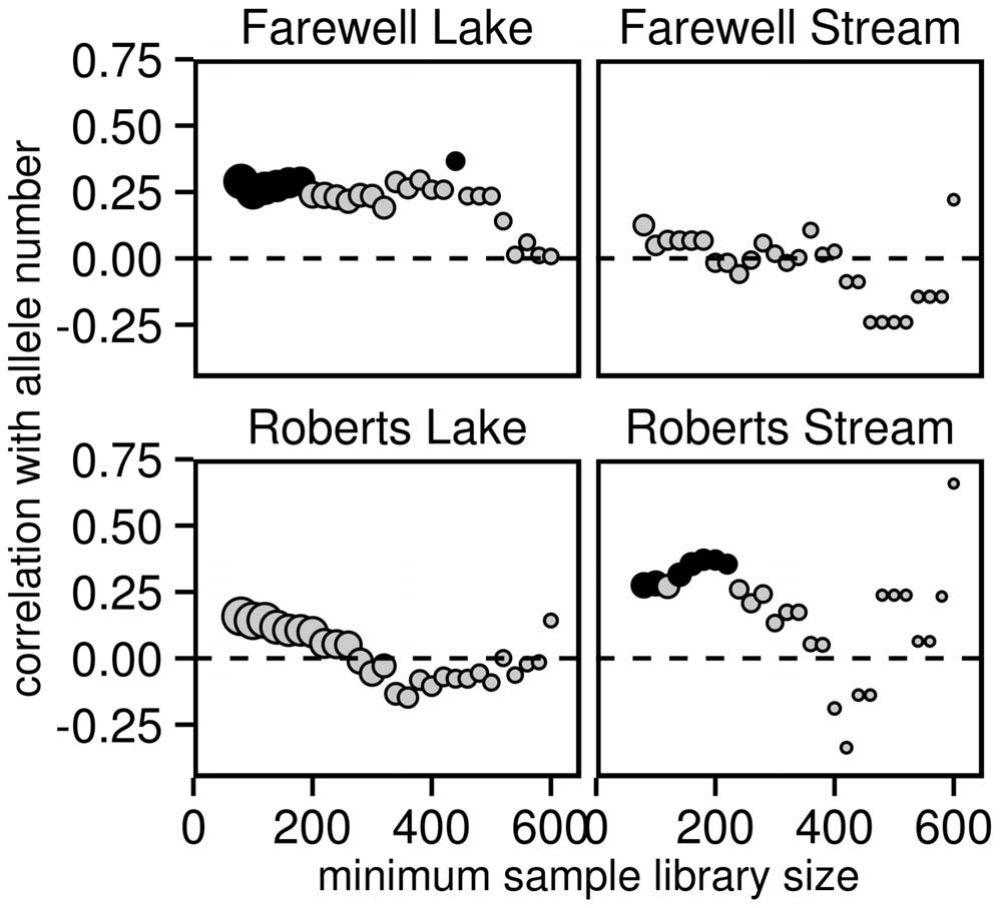
Changes in the correlation between library size and allele number with different minimum sample library sizes. The correlation coefficients shown in figure 5 were recalculated at increasing minimum sample library sizes. Black circles denote statistically significant (α = 0.05) correlations, whereas gray circles denote statistically insignificant correlations. The dotted line indicates a correlation of zero. The size of the circles is proportional to the sample size for each correlation.

To determine whether increasing the minimum sample library size could eliminate the correlations between library size and allele number we repeated the above correlation estimations for all four populations using a range of minimum samples sizes (80 to 600 reads). In all populations, the correlation coefficient decreased as the minimum sample size increased, although the extent and rate of decrease varied among populations (Fig. 6). For both Roberts Stream and Farewell Lake, the correlations remained above r = 0.25 and were significant up to ∼200 read minimum, at which point the correlations began to drop and become statistically indistinguishable from zero. In the other two populations correlations were lower than 0.25 and remained statistically insignificant no matter the minimum library size used. Overall, these results suggest that, at less than 200 reads, some samples may be missing alleles, but that above 200 reads the bias introduced by sample library size was likely reduced.

### Statistical comparisons between populations

Both population pair (Roberts vs. Farewell, F = 6.99, df = 1, P = 0.008) and habitats (lake vs. stream, F = 9.53, df = 1, P = 0.002) differed from each other in the mean number of alleles per individual fish (Fig. 3). In particular, the Roberts fish had, on average, 0.53 fewer alleles than Farewell fish (6.93 vs. 6.39), while stream fish had, on average, 0.67 fewer alleles than lake fish. The interaction between pair and habitat was not significant (F = 0.22, df = 1, P = 0.64), indicating that magnitude of the difference between lakes and streams did not depend on the pair.

**Figure 7:**
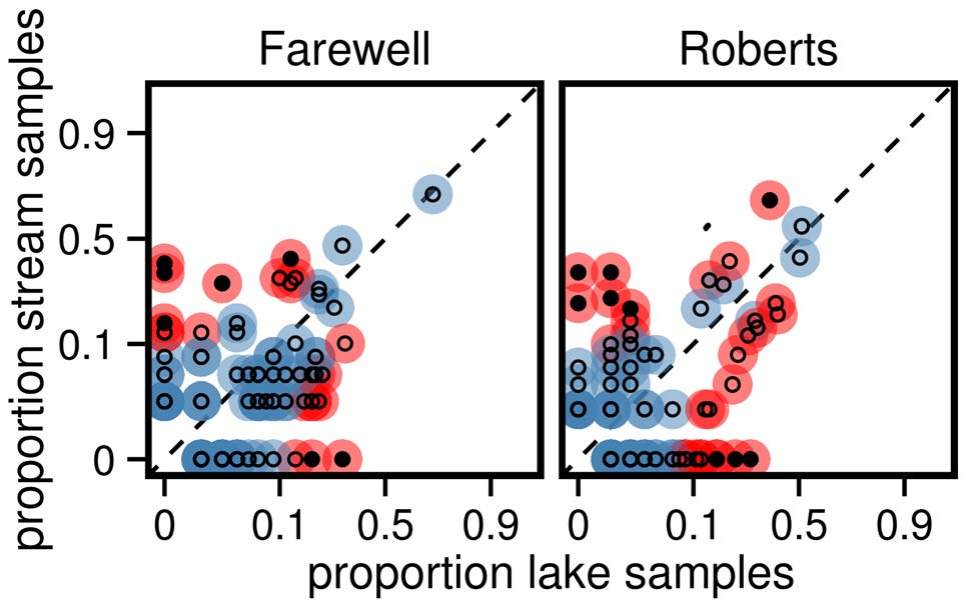
Differences in allele frequencies between lakes and streams. Allele frequency is calculated as the proportion of individuals carrying each allele in each population. Points closer to the dotted line indicated alleles with frequencies similar in the lake and stream populations. Red circles indicate alleles that were significantly different in frequency between lake and stream habitats (within pairs). Blue circles indicate insignificant differences in frequency. Filled black circles indicate that the allele was significantly different after controlling for multiple comparisons (false discovery rate = 5%). Note that frequencies are plotted on a logit (non-linear) scale.

Our populations also differed from each other in their overall MHC allele compositions (Fig. 7). Our two pairs were marginally significantly different from each other (wald = 9.73, P = 0.08) while lakes and streams were clearly significantly different in MHC allele composition (wald = 15.5, P<0.001). There was also a significant interaction between pair and habitat (wald = 6.51, P<0.001), which shows that the alleles were not diverging in frequency between habitats in a parallel fashion among our two pairs.

In addition to the overall significant differences, we also found a number of alleles within each pair that differed significantly in frequency (percentage of individuals where the allele was found) between habitats (Fig. 7, Table S6, S7). In Roberts we found 28 (of 134) alleles that differed significantly in frequency at α = 0.05, of which ten remained significant after controlling for multiple comparisons. Six of these (#40, #265, #347, #336, #103 and #132) were significantly more common in Roberts Lake whereas the other four (#120, #298, #670 and #707) were significantly more common in Roberts Stream. In Farewell we identified 21 (of 128) alleles that were significantly different in frequency between the two habitats, of which seven remain significant after controlling for multiple comparisons. Four were more frequent in the stream (#175, #182, #655 and #659), while the other two (#1850 and #269) were more frequent in the lake. Taken together, these results suggest lake and stream populations have diverged in the relative frequency of certain MHC alleles (sometimes quite substantially) despite a lack of physical barrier to movement between habitats and previous evidence from neutral markers for gene flow between similar paired lake and stream populations [79].

Of the alleles that were significantly different in each pair after controlling for multiple comparisons, our sub-sampling analysis indicated that genotyping only half as many samples would allow us to find the same significant differences between 0 and 91.8% of the time for individual alleles in Roberts (Table S6) and between 0 and 77.1% of the time for individual alleles in Farewell (Table S7). Extending the analysis to alleles that were significantly different before adjusting p-values for multiple comparisons, we found significant differences in as few 0.2% of subsamples in Roberts and 0% in Farewell. Using STC thus appears to provide substantially more power for testing hypotheses about MHC allele frequency differences (and presumably other tests where power can be increased by increased sample size) when compared to methods where only half as many samples can be genotyped, provided the accuracy of the genotyping was roughly equal between the two methods.

## Discussion

Next generation technologies are quickly replacing traditional sequencing and conformation based approaches as the methods most suitable for sequencing multi-locus genes like those of the MHC [51], [52], [80]. In this paper we have proposed a new bioinformatic method for genotyping MHC genes using NGS technology and applied it successfully to a large sample of 295 threespine stickleback. A handful of methods for correctly genotyping MHC using NGS have been proposed in the last few years [44], [45], [53], [80]. These methods have taken standard quality control approaches typically applied to cloning and sequencing and applied them to the output of next generation sequencers. The stated goal of these approaches was to use frequency and similarity criteria to correctly classify sequences as either artifacts or alleles, much as one would with sequences derived from individual clones. STC represents not just a variation or improvement on these methods, but offers a qualitatively different approach to genotyping. STC takes full advantage of the increase in sequence data acquired during next-generation sequencing by using a clustering algorithm to group together similar sequence variants. Because all sequences, artifactual or not, are derived from allelic sequences originally present in the sample, there is information in the artifactual sequences as well that can be used in genotyping alleles.

Previous approaches to genotyping MHC genes using next-generation sequencing technologies have typically struggled with two problems. First, there is a range of read frequencies within which there will be a substantial number of both true allelic sequences and artifactual sequences, making it difficult to adequately balance the rate of false positives and false negatives during genotyping. Second, it can be difficult to distinguish whether two similar, but relatively frequent, sequences represent two alleles or one allele and one artifact derived from that allele. One recent previous approach [53] has proposed solving these problems by running duplicates of every sample, which, while effective, substantially reduces the number of samples that can be successfully genotyped in a given sequencing run. STC is able to overcome these problems in a number of different ways. First, true alleles will usually generate distinct clusters no matter what their frequency (unless they are just a few SNPs away from another true allele), whereas artifacts will generally cluster with the alleles from which they are derived unless they are chimeric or otherwise extremely error ridden. Second, read frequency criteria can be applied within clusters to reliably distinguish clusters that represent two alleles versus one. Note that this discrimination occurs not just during the clustering in phase 3, but is dealt with implicitly in phase 2 during sequence combination, where frequent, but obvious, artifacts are combined with potential alleles based on the error profiles of the given NGS technology. Finally, STC allows the user to be relatively conservative in applying allelic status during phase 3, thus reducing false positives, and then allows the user to recover many of the false negatives that may have dropped out in the subsequent phase 4, if those false negatives are found in other individuals from a population.

### Sample Library Size

One concern of previous authors has been to ensure that enough reads are present in a given sample library to ensure accurate genotyping. Previous methods have taken a probabilistic approach based on multinomial distributions to determine the minimum number of reads required to estimate a genotype at some level of confidence [45]. Sommer et al. [53] take this approach a step further by incorporating estimates of relative amplification efficiency into calculating minimum library sizes necessary to ensure, for example, 99% probability of not missing any alleles. While these recommendations are useful, especially when sequencing an organism for the first time, we believe they are too conservative for at least two reasons. First, Sommer et al. [53] base their recommendations on the maximum expected allele number (in our case 12) and the minimum relative amplification efficiency of any allele to be included in an analysis (for which they provide ways of estimation after genotyping). This effectively targets the worst case scenario and likely overshoots the number of reads necessary to genotype most of the samples in the library, reducing the number of samples that can be genotyped in a given sequencing run. Moreover, the minimum number of reads required is highly dependent of the relative amplification efficiencies, which cannot always be estimated a priori, and for which there is going to be substantial variation between samples.

Second, minimum size recommendations assume that identifying all alleles for all samples is necessary for accurately estimating all the parameters or testing all the hypotheses one might be interested in. This is not necessarily true. If the goal is to provide estimates of population level diversity (i.e. how many alleles are present in the population) having some samples with incomplete genotypes due to having fewer reads is unlikely to change the population estimate. In fact, in such situations it would be better to aim for genotyping more samples than for increasing the number of reads for each sample, which increases the chances of finding rare alleles. Alternatively, if the final goal is to compare individual level diversity with some other measured phenotype (i.e. parasite burden), missing an allele in a few samples may alter the effect size estimate only slightly. In those cases, one possibility would be to use read number as a covariate in downstream analyses, or weight individual allelic diversities by the number of reads. If the final goal is to use the presence or absence of a given allele in association tests with other phenotypes (i.e. parasite infection), a small number of false negatives due to incomplete genotyping will, at worst, reduce statistical power but is unlikely to bias associations one way or the other. Our overall results suggest that having at least 200 reads was sufficient for reducing the bias introduced by small library sizes, although even this number may be too large if we remove the highly divergent, very low efficiency alleles from the analysis. Ideally, we would recommend targeting at least 50 reads per allele for a sample with mean allelic diversity (i.e. ∼target 350 reads per sample if the expected average allele number of 7), or more as funds allow, because there will always be variability in the number of reads per sample in any given run.

### Amplification Efficiency

Even if library sizes are adequate for genotyping, it is possible that some alleles may amplify with relatively lower efficiency, either because of differences in the primer or amplicon sequence, or because of differences in PCR conditions between reactions and runs. STC allows for the identification of such alleles in two ways. First, alleles that amplify with low efficiency will have, on average, lower relative cluster sizes than other alleles. Second, alleles that tend to amplify with low efficiency will be more likely to be dropped during phase 3 before being added back to genotypes during phase 4. We showed that cluster size and rate of dropping in phase 3 were moderately negatively correlated in our data set, and plotting the relationship suggested that a group of highly divergent alleles were especially likely to be dropped (Fig. 4, Fig. 5). As suggested by our results from duplicated samples, it is possible that, in many cases, these alleles were not only dropped during phase 3, but were completely absent from some sample libraries. If the alleles in this group originated from a single divergent locus, it would mean that in 52 of 302 samples we did not identify any alleles from this locus.

One of our divergent alleles (#83) has been previously reported by Lenz et al. [42], who found this sequence in every one of 23 cloned and sequenced three-spine stickleback samples (sequence was previously uploaded to GenBank: AF395709). Similarly, the same sequence was found in a highly conserved cluster of similar sequences in a sample of 30 threespine stickleback and in a sample of 30 nine-spine stickleback (*Pungitius pungitius*) [33]. These authors speculated that these sequences, because of their lack of diversity among individuals, could potentially originate from an invariant MHC class-II like locus involved in antigen processing [81]. Blasting these sequences against the published stickleback genome [61] reveals that they all clearly map to a single MHC class II locus (located on linkage group VII) with associated expressed sequence tags (ESTs). Using a comparative approach among teleosts, Dijkstra et al [60] argue that this locus is, in fact, a classical MHC class II locus (DB type) and not an MHC class II-like locus (DM type) that are thought to be involved in antigen processing. In our data set, this cluster of divergent alleles share, on average, only 65% sequence similarity with other class II alleles, contain no frame shifts (except in one case) or premature stop codons, and retain enough of the forward and reverse primer sequences to be amplified in most cases. However, without further information it may be difficult to determine whether these alleles are traditional class II or class II-like alleles, but no evidence presented here would suggest they do not originate from a normal MHC class II locus. One possible explanation for their lack of genetic diversity is that their isolated genomic location relative to other MHC class II loci [60] may not predispose them to gene conversion or recombination events, which is thought to be one of the primary generators of MHC allelic diversity [35], [82], [83].

### Single Read Alleles

Aside from stochastic or amplification bias, cases where alleles are represented by only one or two reads could also result from sequencing errors in the barcodes such that reads are assigned to the incorrect sample (fairly unlikely given the redundancy of our barcodes). Alternatively, a very small amount of contamination during PCR setup could introduce alleles from other samples into the PC template. Previous approaches to genotyping have required at least three reads of a given sequence in at least two samples to consider it as a possible true allele [45], [46]. In contrast, STC includes no such a priori filter. Although clusters must pass a size threshold before being considered good clusters (and thus true alleles), STC includes a cross-checking step that allows users to include small clusters as true alleles if they appear in larger clusters in other samples. In many cases this will result in a decrease in false negatives, because alleles that amplify at with low efficiency will often be represented by only a few reads, especially in sample libraries of small to moderate size. In theory, small clusters represented by only a single read could be included as true alleles. We leave it to the user to decide how best to deal with such cases. We have taken the conservative approach of only adding small clusters as dropped true alleles if the total number of reads in the cluster is at least three. This is superficially similar to previous approaches, but note that only the cluster must contain at least three reads but that the reads do not have to represent the same sequence. Note also that, in STC, a sequence could be represented by two reads and be combined with a number of other sequences during phase 2 to increase its total read count well above three. An alternative approach would be to omit single read and double read clusters only when they do not represent low-efficiency alleles (as defined by the user based on average cluster sizes or frequency of dropping out during phase 3). A more probabilistic approach could, after phase 4, use multinomial probability distributions to omit clusters that would not occur in 95% of sequencing runs, assuming the same library size and number of alleles.

### Alternative Sequencing Technologies

An important feature of STC is that it can be applied, in theory, to any data set of MHC allele sequences (or other multi-allelic amplicon from a gene family) generated by NGS technology as long as 1) many reads can be generated for individual samples and 2) individual sample libraries can be subset from the entire library using barcoding or other techniques. As other sequencing technologies increase their average read lengths, researchers will likely begin to shift away from pyrosequencing to alternative technologies (i.e. Illumina). There are a number of issues to consider when applying STC to data sets generated using such alternatives. First, Illumina sequencing typically produces many more reads per sequencing run than does pyrosequencing. However, sequencing facilities can often target a very specific number of sequences with barcoding, and thus users should be able to specify a smaller target number of reads based on the number of samples and desired coverage. Second, Illumina sequencing is more prone to substitution errors than to indel errors. In phase 2 of STC, sequences are combined when they are very similar but of different lengths, meaning with Illumina data few sequences may be combined. Future implementations of STC for Illumina data could skip phase 2 entirely, or phase 2 could be modified to combine sequences where a single substitution results in the creation of an open reading frame (i.e. a stop codon). Third, error rates tend to be an order of magnitude lower (0.3–4% versus 12%) than with pyrosequencing. This would mean that the proportion threshold (γ) will likely need to increased to something around 9∶1 rather than 4∶1 as currently implemented for pyrosequencing. One of the overall advantages of STC is its flexibility, giving the user the ability to adjust the STC parameters based on the number of samples, the number of amplified loci, and expected error rates. Although we have not yet implemented STC for non-pyrosequenced data, we see no reason why STC could not be applied directly to Illumina generated sequence data with minimal modifications to the STC protocol.

### Future Directions

One of the advantages of STC is that all the reads present in the sample library contribute to genotyping. This could provide a number of potential advantages beyond simply genotyping. First, by grouping reads into clusters that derive from true alleles, a better estimate of the relative amplification efficiencies could be estimated than if relative efficiencies are estimated only from reads that match alleles directly [45], [53]. Second, current methods do not allow for estimation of allele and locus copy number within samples. Alleles may be present as homo or heterozygotes, or may be present at more than one locus due to gene duplication [25], [32], [37]. The number of loci may also vary between individuals, even within a single populations [16]. Currently, only the presence or absence of alleles can be inferred when loci are amplified simultaneously, and only a maximum MHC locus number can be inferred from the most diverse genotyped samples. It is possible that the data provided by grouping reads into clusters could be used in a full probabilistic model to simultaneously infer amplification efficiencies, allele copy, and locus copy number across all samples simultaneously. Such a model is beyond the scope of this paper, but we note that Dirichlet processes have been used as priors in Bayesian models infer HIV haplotype number from DNA isolated from infected patients [84], [85]. We believe the success of these models suggest that STC, or similar, recently introduced methods [86], may, in the future, provide researchers not only with high-throughput genotyping of MHC in non-model organisms, but provide a fuller picture of multi-locus MHC genotypes as well. For the present we offer STC as an efficient, accurate, and extremely flexible method for genotyping MHC (and other multi-locus templates) using NGS which produces data that can be applied to a variety of downstream parameter estimation and hypothesis testing applications.

## Supporting Information

Figure S1 **Negative correlation between average good cluster size and frequency of dropping.** Each point indicates a single allele. Calculation of cluster size averages do not include clusters originally dropped in phase 3. Each point indicates a genotyped sample. Samples with more than 1000 reads were sub-sampled to 1000 reads. No B (duplicate) samples were included to avoid pseudo-replication. he solid line indicates the best-fit linear regression. The 95% confidence band for the regression is indicated in gray. Alleles associated with the “divergent” allele cluster (see Figure S2) are filled in black.
(TIFF)

Figure S2 **Unrooted, neighbor-joining tree of all alleles appearing in at least 10 samples.** Dotted rectangle highlights group of highly divergent sequences (see also Table S5), all of which appear to amplify with lower efficiency than most other alleles. Three other alleles (two singletons) also belong in also group with these alleles, but were present in fewer than 10 samples. Scale bar indicates 1% sequence similarity (∼2 bp).
(TIFF)

Figure S3 **Correlations between good cluster number and minimum library size.** Plots are identical to figure 5, except that the y-axis shows only alleles identified through phase 3 (i.e. good, but not dropped, clusters). Samples with more than 1000 reads were sub-sampled to 1000 reads. Points represent individual genotyped samples. No B (duplicate) samples were included to avoid pseudo-replication. The lines indicate the best-fit linear regressions for each population. The confidence bands for each regression are indicated in gray.
(TIFF)

Table S1 **List of barcodes used for 454 sequencing.**
(XLS)

Table S2 **Genotyping results for individual samples.**
(XLS)

Table S3 **Clustering results for individual sequences from sample X.**
(XLS)

Table S4 **Combined pairs of sequences from sample X.**
(XLS)

Table S5 **Table of alleles identified across all samples.**
(XLS)

Table S6 **Allele frequencies, G-tests, and sub-sampling analysis results (Roberts).** G-statistics are from tests of whether the lake and stream differ significantly in allele frequency for each given allele. The proportion of sub-samples where sample size was divided in half (total = 10000) where the allele was statistically significant between habitats (α = 0.05) is given. Both p-values and proportion of significant sub-samples are shown when p-values are unadjusted and adjusted using a false discovery rate of 5%.
(XLS)

Table S7 **Allele frequencies, G-tests, and sub-sampling analysis results (Farewell).** G-statistics are from tests of whether the lake and stream differ significantly in allele frequency for each given allele. The proportion of sub-samples where sample size was divided in half (total = 10000) where the allele was statistically significant between habitats (α = 0.05) is given. Both p-values and proportion of significant sub-samples are shown when p-values are unadjusted and adjusted using a false discovery rate of 5%.
(XLS)

File S1 **R script (runSTC.R) for running phases 2–4 of STC.** This script (along with the accompanying functions, will allow users to run phases 2–4 of STC. A more thorough version of this file is available at the Dryad Digital Repository (http://dx.doi.org/10.5061/dryad.4fn4g), along with a README.txt file that contains more information on using the scripts and example data files.
(R) Click here for additional data file.

File S2 **R functions to accompany the runSTC.R script (File S1).**
(R) Click here for additional data file.
