# Supplementary figures and images for: Stepwise Threshold Clustering: A New Method for Genotyping MHC Loci Using Next-Generation Sequencing Technology
Source: PLoS One. 2014 Jul 18;9(7):e100587. doi: 10.1371/journal.pone.0100587 (PMC4103772; doi:10.1371/journal.pone.0100587)

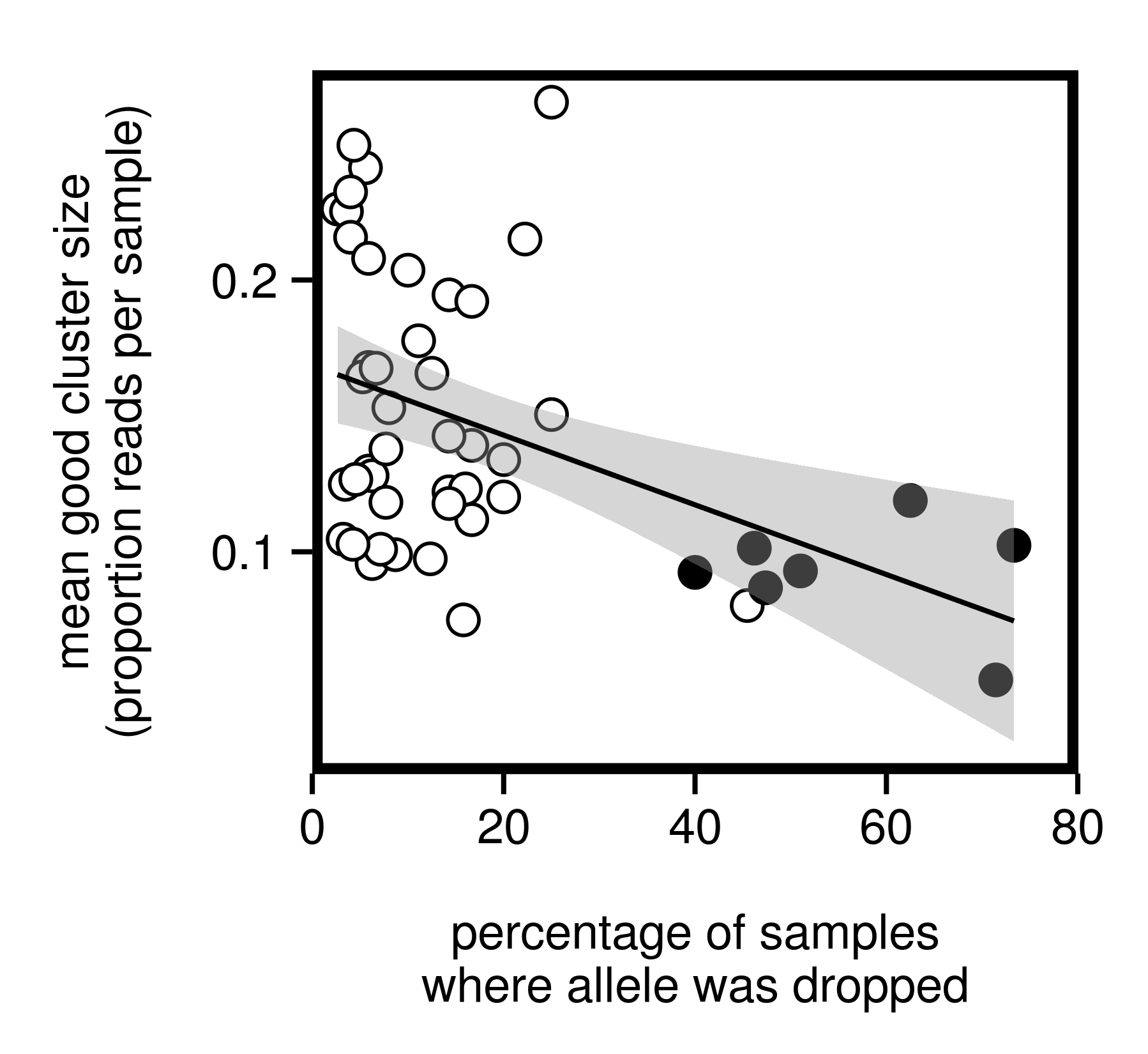

Supplement: Figure S1 — Negative correlation between average good cluster size and frequency of dropping. Each point indicates a single allele. Calculation of cluster size averages do not include clusters originally dropped in phase 3. Each point indicates a genotyped sample. Samples with more than 1000 reads were sub-sampled to 1000 reads. No B (duplicate) samples were included to avoid pseudo-replication. he solid line indicates the best-fit linear regression. The 95% confidence band for the regression is indicated in gray. Alleles associated with the “divergent” allele cluster (see Figure S2) are filled in black. (TIFF) [file pone.0100587.s001.tiff]

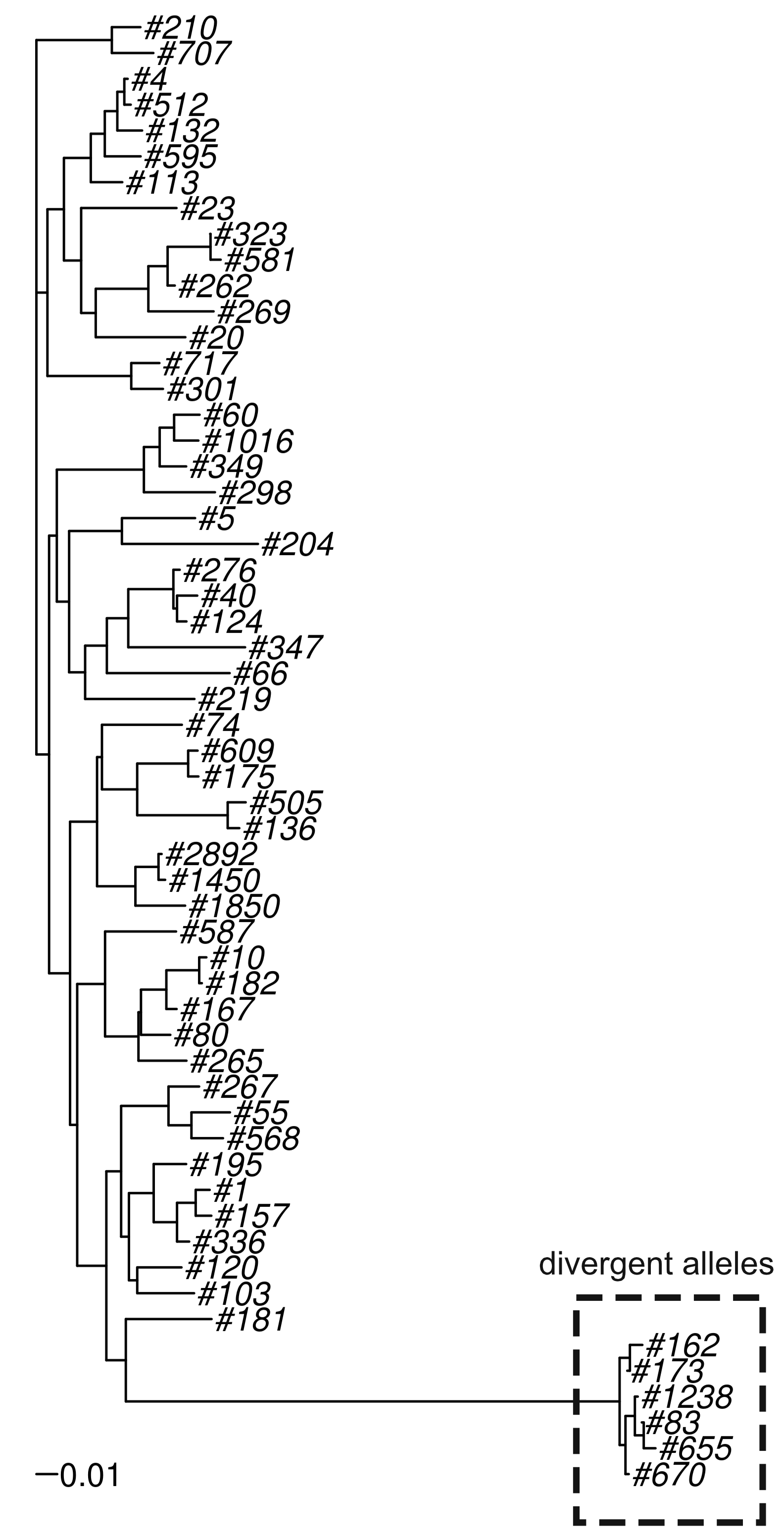

Supplement: Figure S2 — Unrooted, neighbor-joining tree of all alleles appearing in at least 10 samples. Dotted rectangle highlights group of highly divergent sequences (see also Table S5), all of which appear to amplify with lower efficiency than most other alleles. Three other alleles (two singletons) also belong in also group with these alleles, but were present in fewer than 10 samples. Scale bar indicates 1% sequence similarity (∼2 bp). (TIFF) [file pone.0100587.s002.tiff]

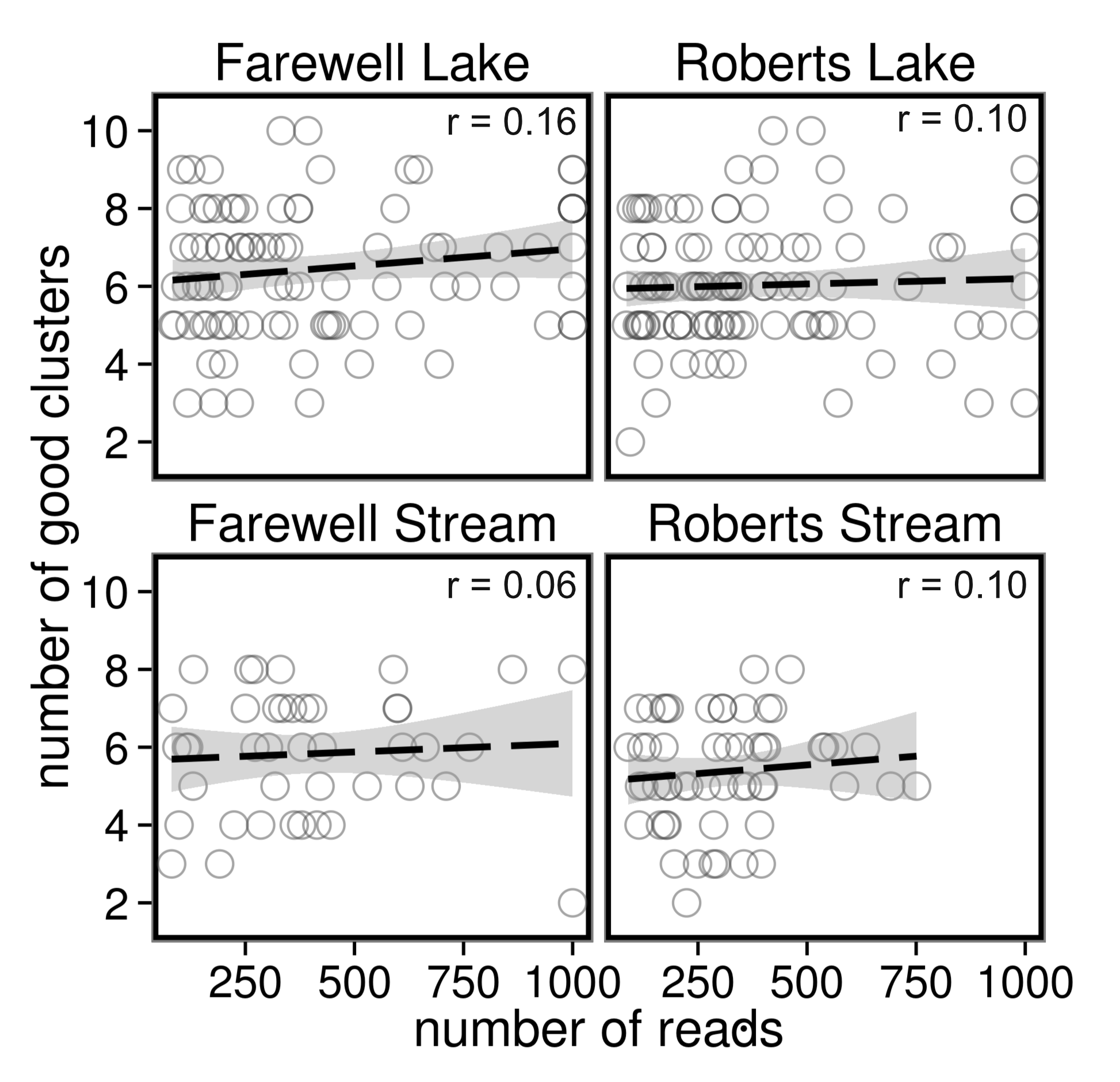

Supplement: Figure S3 — Correlations between good cluster number and minimum library size. Plots are identical to figure 5, except that the y-axis shows only alleles identified through phase 3 (i.e. good, but not dropped, clusters). Samples with more than 1000 reads were sub-sampled to 1000 reads. Points represent individual genotyped samples. No B (duplicate) samples were included to avoid pseudo-replication. The lines indicate the best-fit linear regressions for each population. The confidence bands for each regression are indicated in gray. (TIFF) [file pone.0100587.s003.tiff]
